# Profiling rhythmicity of bile salt hydrolase activity in the gut lumen with a rapid fluorescence assay†

**DOI:** 10.1039/d2ob02257e

**Published:** 2023-02-18

**Authors:** Chathuri J. Kombala, Neha Agrawal, Agne Sveistyte, Ilia N. Karatsoreos, Hans P. A. Van Dongen, Kristoffer R. Brandvold

**Affiliations:** a Biological Sciences Division, Pacific Northwest National Laboratory Richland WA 99352 USA krbrandvold@gmail.com; b Department of Translational Medicine and Physiology, Elson S. Floyd College of Medicine, Washington State University Spokane WA 99202 USA; c Department of Psychological and Brain Sciences, University of Massachusetts Amherst MA 01003 USA; d Sleep and Performance Research Center, Washington State University Spokane WA 99202 USA; e Department of Nutrition and Exercise Physiology, Elson S. Floyd College of Medicine, Washington State University Spokane WA 99202 USA

## Abstract

Diurnal rhythmicity of cellular function is key to survival for most organisms on Earth. Many circadian functions are driven by the brain, but regulation of a separate set of peripheral rhythms remains poorly understood. The gut microbiome is a potential candidate for regulation of host peripheral rhythms, and this study sought to specifically examine the process of microbial bile salt biotransformation. To enable this work, an assay for bile salt hydrolase (BSH) that could work with small quantities of stool samples was necessary. Using a turn-on fluorescence probe, we developed a rapid and inexpensive assay to detect BSH enzyme activity with concentrations as low as 6–25 μM, which is considerably more robust than prior approaches. We successfully applied this rhodamine-based assay to detect BSH activity in a wide range of biological samples such as recombinant protein, whole cells, fecal samples, and gut lumen content from mice. We were able to detect significant BSH activity in small amounts of mouse fecal/gut content (20–50 mg) within 2 h, which illustrates its potential for use in various biological/clinical applications. Using this assay, we investigated the diurnal fluctuations of BSH activity in the large intestine of mice. By using time restricted feeding conditions, we provided direct evidence of 24 h rhythmicity in microbiome BSH activity levels and showed that this rhythmicity is influenced by feeding patterns. Our novel function-centric approach has potential to aid in the discovery of therapeutic, diet, or lifestyle interventions for correction of circadian perturbations linked to bile metabolism.

## Abstract 2

24-hour rhythmicity in bile salt hydrolase activity levels is influenced by feeding patterns, providing a pathway for the gut microbiome to affect host peripheral circadian rhythmicity.

## Graphical abstract

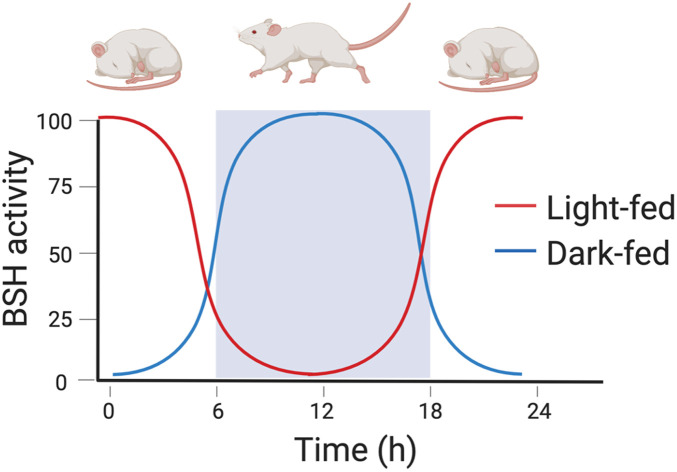

## Introduction

In industrialized nations, many professions (first responders, health care providers, military, *etc*.) require irregular sleep schedules with extensive nighttime activity, or frequent travel across time zones, both of which lead to misalignment between behavioral cycles and the biological clock.^1–5^

Circadian misalignment between the master biological clock and peripheral organs, such as the gut,^6–8^ liver,^9–11^ and pancreas,^10–12^ leads to a desynchrony that disrupts metabolic processes, which can increase susceptibility to the eventual development of metabolic diseases including obesity,^12,13^ type 2 diabetes,^12,14,15^ and cardiovascular disease.^15,16^ Amelioration of the effects of circadian misalignment would improve personal well-being and decrease cost to society, but the mechanisms through which externally imposed behavioral schedules can dysregulate rhythms in metabolic organs are not currently known. The two main external factors that could produce desynchrony are sleep/wake cycle^1,17^ and the feeding/fasting schedule.^18,19^ Sleep deprivation alone does not invoke desynchronization,^18,20^ so investigating the impact of altered feeding/fasting schedules is particularly appealing. One possibility is that microbes in the gut create signals based on the feeding schedule^20–24^ which, in turn, regulate peripheral circadian rhythms in the host that are autonomous from the master clock in the brain.^25–28^ Nutrient availability directly affects microbiome composition^28–30^ which may act as a timing signal for peripheral circadian rhythms, which is consistent with the observation that certain taxa bloom directly following food ingestion, while others become dominant between meals.^31,32^ If eating schedules are erratic or not in-sync with the central clock, misalignment could occur. In order to understand why individuals with irregular sleep patterns are predisposed to metabolic disorders^33,34^ therefore, the microbiome's role in the circadian desynchronization must be appreciated. Promisingly, the gut microbiome is amenable to reshaping, and some characteristics can be rapidly impacted, which makes the gut microbiome an appealing target for therapeutic correction of circadian misalignment.^35–38^

While sequence-based analyses infer functional rhythmicity based on taxa abundances, direct measurement of microbiome function is under-reported. The extent of functional rhythmicity of the microbiome must be established to understand interactions with host rhythms. We became interested in the possibility of rhythmicity of microbial bile metabolism after we developed a novel assay to quantify the activity of a key metabolic enzyme. Bile is a host-produced fluid that enables the digestion of dietary fats and some lipophilic vitamins, due to the presence of amphipathic bile acid (BA) molecules.^39^ Microbial BA metabolism directly influence host physiology through changing the capacity to bind host receptors as signaling molecules,^40^ which impacts the regulation of host dietary lipid absorption,^41–43^ energy homeostasis,^44^ cholesterol metabolism,^41^ and controlling secondary BA level.^45,46^ Since bile is released from the gall bladder at feeding times, this could act as a “zeitgeber” for peripheral rhythms associated with bile metabolism.

Metabolomics data have already suggested that the bile salt pool experiences daily fluctuations,^47^ but confounding factors such as changes in bile salt levels due to variations in liver production or excretion in feces could influence the final measurements of metabolomics-based assays. A different approach is needed to investigate rhythmicity in bile metabolism. Multiple structural modifications may occur on a single BA molecule from microbial metabolism, but the first step is always bile salt hydrolysis, which results in free amino acid and BA products (Fig. S1A†). The enzyme responsible for deconjugation, BSH, is therefore thought of as a gatekeeper of microbial BA biotransformation.

Characterization of BSH rhythmicity, and determination of regulatory factors, has been previously untenable due to a lack of tools to specifically report on BSH activity in laboratory-relevant quantities, and issues that plague the study of microbiome functions in general, including inadequate genome annotation. For example, the Unified Human Gastrointestinal Protein catalog reported that there are 204 938 nonredundant genomes recorded from human gut microbiota, with 71% of total species having no experimental characterization and 40% no functional annotation whatsoever.^48^ Therefore, the likelihood of overlooked or improperly annotated BSHs in conventional omics measurements is high. Even in an ideal scenario, with a hypothetically fully annotated microbiome metagenome, transcript measurements do not confirm protein presence, and methods that monitor protein abundance cannot confirm that the enzymes are active, which may be influenced by secondary factors such as co-factor and substrate concentrations, cellular localization, protein–protein interactions, and post-translation modifications. As such, methods for functional measurement must be prioritized.

We sought to determine unambiguously if BSH activity is rhythmic and provide information on potential regulatory factors through imposition of a feeding schedule that emulates shift work. Direct evaluation of BSH activity in intestinal samples is very challenging due to the complex biological environment. Current methods of assessing BSH activity involve chromatographic techniques such as HPLC which require expensive instrumentation and long assay times.^49^ A spectrophotometric assay has been developed recently using bioluminescence probes^50^ which requires complex experimental setup as additional luciferase enzyme or luciferase transfected cells are needed for the assay. We also realized that our previously reported fluorogenic BSH assay,^51^ which required long incubation periods and large amount of sample, would be impractical in circadian studies due to inherently larger numbers of samples from multiple data collection time points. Therefore, we first sought to develop a novel fluorogenic substrate, which we refer to as ChoRhoS (CholoylRhodamine Substrate), which is a substantial improvement over our previous approach,^51,52^ because the red-shifted rhodamine fluorophore has lower background noise. In addition, with new assay conditions we reduced the assay time (30× faster) and increased our signal (20-fold reduction in sample size) and only of fraction of a mouse fecal pellet would be needed for analysis. Collectively, these improvements allowed us to definitively characterize the rhythmicity of BSH activity.

### Chemical synthesis and spectroscopic characterization of ChoRhoS and related probes

**Fig. 1:**
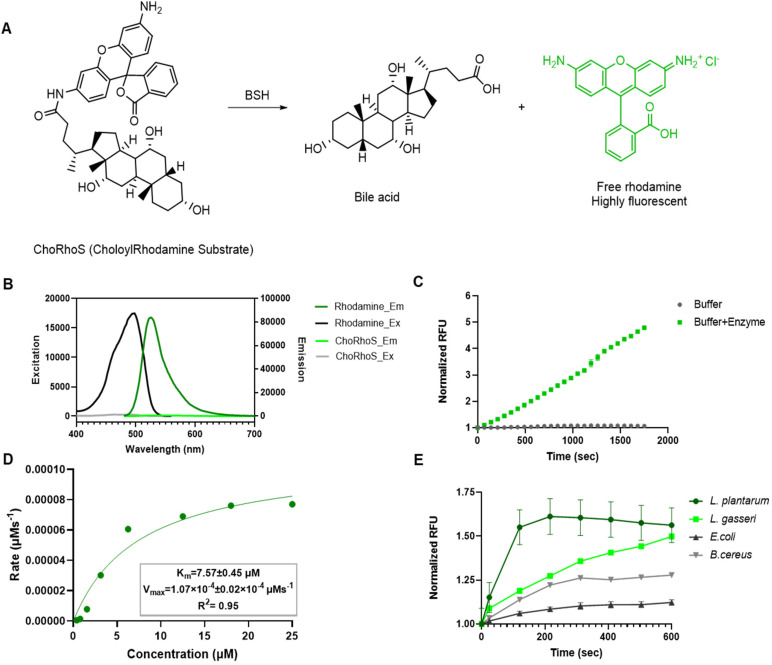
Preliminary characterization of the synthetic CholoylRhodamine Substrate (ChoRhoS). (A) Fluorogenic BSH substrate was developed by conjugating rhodamine to cholic acid. Upon exposure of synthetic substrate to BSH, the amide bond is hydrolyzed and produces a free BA and free rhodamine, which is a highly fluorescent compound compared to its conjugated version. (B) Spectral properties for ChoRhoS. Solutions of rhodamine 110 (25 μM) and ChoRhoS (25 μM) in PBS were characterized to determine excitation and emission properties of substrate and the cleaved product. Fluorescence of free rhodamine was significantly higher than ChoRhoS (*p* < 0.0001) at the same concentration. Emission spectra were recorded at 495 nm excitation wavelength and excitation spectra were recorded at 525 nm emission wavelength. (C) A solution of 25 μM ChoRhoS in pH 6.0 PBS was incubated with 40 μg mL^−1^ purified BSH, and product formation was observed. (D) Buffered solutions of 40 μg mL^−1^ purified BSH were treated with varying concentrations of substrate. The kinetic parameters for ChoRhoS hydrolysis by BSH were determined using a Michaelis–Menten analysis. (E) Probe cleavage observed in BSH expressing monocultures (*Lactobacillus plantarum* (*L. plantarum*) and *Lactobacillus gasseri* (*L. gasseri*)) was significantly higher than in non-BSH expressing monocultures (*Escherichia coli* (*E. coli*) and *Bacillus cereus* (*B. cereus*)) at the same cell density. ChoRhoS (25 μM) was co-incubated with cell suspensions. All biochemical reactions were carried out at 37 °C, and probe hydrolysis was quantified by measuring fluorescence at 495 nm and 525 nm excitation and emission wavelengths using a plate reader.

For our BSH assay, we employed an approach commonly used for amide hydrolases that is based on evolution of rhodamine fluorescence. Acylation of rhodamine nitrogen atoms locks the molecule into a closed lactone form that is less fluorescent or nonfluorescent,^53^ and this enables a variety of fluorogenic enzyme substrates to be built from *N*,*N*′-mono or diacylrhodamines, which produce a highly fluorescent free dye upon exposure to an amide cleaving enzyme. Thus, we designed synthetic substrates (Fig. S1B†), which are conjugation products of cholic acid and rhodamine. The designed substrate contains an amide bond that is specifically cleaved by the BSH enzyme (Fig. S1C†), which results in evolution of fluorescence that is proportional to the level of BSH activity (Fig. 1A). We conjugated cholic acid with rhodamine using standard EDC coupling reagents with dimethylformamide (DMF) as a solvent and DIEA as a base (Scheme S1†). The conjugated product was isolated *via* flash chromatography. Spectroscopic characterization of the conjugated product and free rhodamine showed at least 130-fold less fluorescence for the substrate relative to free rhodamine (Fig. 1B and S1D, Table S1†). This is substantially higher than the 10-fold increase observed with our earlier coumarin-based substrates. Therefore, our probe showed potential for use as a fluorogenic reporter in BSH activity assays.

## Results

### Biochemical characterization of ChoRhoS with purified recombinant BSH enzyme

We first tested our probe with purified, recombinant *Lactobacillus plantarum* (*L. plantarum*) BSH. When the mono-cholic conjugate probe (ChoRhoS) was exposed to purified recombinant BSH, rhodamine fluorescence increased significantly (Fig. 1C-green). To confirm the necessity of the cholic acid moiety, we synthesized *N*-acetylrhodamine as a control substrate, and no fluorescence increase was observed upon incubation with recombinant BSH enzyme (Fig. S1E†). Therefore, the evolution of fluorescence due to co-incubation of BSH with ChoRhoS is not due to non-specific amide cleavage. We also confirmed that the probe was not simply degrading *via* hydrolysis. No increase in fluorescence was observed when the probe was added just to buffer, indicating the stability of the probe in buffered solutions (Fig. 1C-grey). We were able to detect fluorescence signals from BSH-promoted substrate turnover in the low micromolar concentration range for the substrate, which is a significant improvement over our previous assay with coumarin-derived fluorogenic substrates, which we typically used at a final concentration of 150 μM. We also synthesized di-cholic acid conjugated rhodamine (Di-ChoRhoS; Fig. S1B†) and tested with recombinant protein. Surprisingly, no cleavage was observed for Di-ChoRhoS (Fig. S1E†-gray), which could be due to the steric hindrance from two cholic acid moieties. Due to the negligible activity, Di-ChoRhoS was dropped from consideration, and further probe characterization focused on ChoRhoS.

We then quantitatively examined the kinetics of ChoRhoS hydrolysis by recombinant BSH *via* a standard Michaelis–Menten analysis. Our biochemical characterization of ChoRhoS revealed a low micromolar Michaelis–Menten constant (*K*_M_ = 7.57 μM; Fig. 1D and S2†), which is substantially lower than reported values^54^ for native BSH substrates and 8-fold lower than our previously reported synthetic BSH fluorescence probes. The *V*_max_ was observed as 1.07 × 10^−4^ s^−1^, and the catalytic efficiency (*k*_cat_/*K*_m_) for the enzyme was 1.32 M^−1^ s^−1^.

### Whole-cell characterization of ChoRhoS

After confirming that the ChoRhoS probe is an effective fluorogenic reporter for recombinant BSH, the possibility of analyzing complex biological mixtures was first evaluated with whole-cell suspensions. We tested BSH positive organisms (*L. plantarum* and *L. gasseri*) and BSH negative organisms (*E. coli* Nissle and *B. cereus*) and observed a correlation between fluorescence evolution and the BSH activity of whole cell monocultures (Fig. 1E and S3, S4†), which suggests that our probe selectively detects activity in BSH expressing organisms (Fig. S3–S5†). Additionally, we observed that fluorescence was proportional to cell density in a dilution series of *L. plantarum* cells (Fig. S3A†). Finally, we established that ChoRhoS could accurately measure BSH activity in monoculture mixtures (Fig. S3B†), suggesting that the assay could work with more complex samples.

### Mouse fecal sample analysis using ChoRhoS

**Fig. 2:**
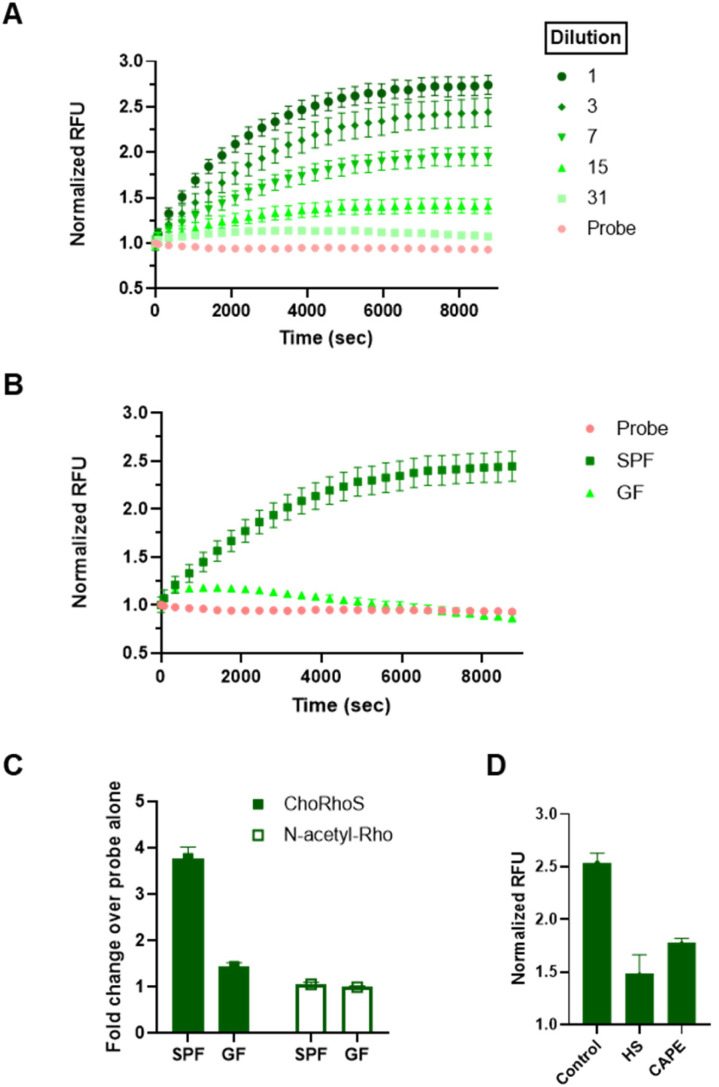
Characterization of ChoRhoS probe in mouse fecal microbiome samples. Mouse fecal samples were suspended in PBS with 20 mM beta-mercaptoethanol (BME) with a concentration of 50 mg mL^−1^. Fecal suspensions were then serially diluted to obtain different cell densities. (A) Fluorescence evolution resulting from hydrolysis of ChoRhoS probe (25 μM) correlates with density of conventional (specific pathogen-free; SPF) C57BL/6J mouse fecal suspensions at 37 °C. (B) Comparison of normalized RFU kinetic plots for fecal suspensions from SPF and germ-free (GF) C57BL/6J mice at 1 : 1 sample density showed a significant difference (*p* < 0.0001). (C) After 2 h of probe incubation, an increase in probe fluorescence was only observed with the ChoRhoS probe, and *N*-acetylrhodamine did not show an increase in fluorescence, indicating BSH specifically cleaves the cholic acid conjugated rhodamine substrate. (D) SPF fecal suspensions were pre-treated to lower the BSH activity through heat-shock (HS; 95 °C for 1 h) and with a BSH inhibitor, caffeic acid phenethyl ester (CAPE; 200 μM). RFU values were normalized to the RFU at *t* = 0 (initial fluorescence reading). Relative fluorescence unit (RFU) values after HS (*p* < 0.0005) and BSH inhibitor (*p* = 0.0014) treatment were significantly lower compared with untreated fecal suspensions.

Detecting BSH activity in fecal samples is challenging due to complex and highly heterogeneous sample composition, which is compounded by the limitations of current methods that require extensive sample processing, long assay time, and complex experimental setup.^50^ Our previous approach, required overnight incubation for any observable signal of native BSH in fecal samples relative to controls. For our new, more rapid approach, we processed fecal samples from conventional (specific pathogen-free; SPF) mice as described previously,^50^ with minor modifications. After addition of the probe to fecal suspensions, we monitored the kinetics of rhodamine liberation for 3 h. Substantial signal evolution was observed within 1 h, and a substrate turnover plateau was observed within 2 h (Fig. 2A and S6†). In comparison with our previously reported fluorescence assay (cholic acid-conjugated amino coumarin probe; CA-AMCA), which requires 1000 mg of mouse fecal sample for analysis, this assay can be effectively applied to detect BSH activity with 20–50 mg of sample. In addition to the improved characteristics of our probe, we also found that the addition of β-mercaptoethanol, which is commonly added as a reductant to maximize the activity of cysteine hydrolases, including BSH,^50^ increased assay signal. These conditions improved the activity of our previously published coumarin-based substrates as well (Fig. S7†), but the ChoRhoS assay still performed substantially better under the same conditions. The S/N ratio for ChoRhoS substrate turnover was around 3-fold higher than the cholic acid-conjugated aminocoumarin probe.

To demonstrate that the observed signal was the result of microbial activity, we incubated our probe with fecal suspensions of feces from germ-free (GF) mice, with the expectation that we should not see appreciable signal in these samples. As expected, a negligible fluorescence increase was observed in fecal suspensions from GF mice relative to the background fluorescence from the probe alone, which contrasts with the significant increase in fluorescence that was observed within 2 h for fecal suspensions from SPF mice (Fig. 2B and C).

To confirm that the observed activity in SPF samples was from properly folded and active enzymes, we conducted additional experiments in which a significant reduction in evolution of fluorescence was detected when fecal slurries were heat-shocked or incubated with a BSH inhibitor, caffeic acid phenethyl ester (CAPE), prior to addition of the probe (Fig. 2D and S8, S9†). We also confirmed that the observed activity in fecal samples was not due to nonspecific degradation of amides through co-incubation of fecal suspensions with a control probe, *N*-acetylrhodamine, which, similar to our bile salt probes, has the amine of rhodamine in an amide bond, but does not have the BA element. In contrast to the ChoRhoS probe, no signal increase upon addition to a fecal sample was observed with *N*-acetylrhodamine (Fig. 2C and S10†); in fact, a slight decrease in signal relative to the buffer was observed. Collectively, our results strongly suggest that the observed signal using ChoRhoS is specifically from active BSH.

The encouraging observations from our mouse fecal sample analysis prompted us to determine if this would additionally translate to the analysis of human fecal samples. In similar experiments, we observed probe fluorescence upon incubation of different dilutions of human fecal suspensions (Fig. S11†). However, although there was a statistically significant difference relative to controls (Fig. S12†), we only observed a 1.04-fold increase in fluorescence for human samples, which is substantially less than the 2.25-fold increase in mouse samples (Fig. S9B†). The lower intensity in human samples could be due to human samples being more heterogeneous as a result of a more complex diet. Thus, further optimization of assay conditions is needed for robust analysis of human samples.

### Evaluation of BSH activity in gut luminal content using ChoRhoS

While fecal samples offer a convenient, noninvasive means of characterizing the gut microbiome, microbial activity in the actual gut lumen of a living animal cannot be fully captured by this approach, which is important in the context of BSH activity because this is where free BA products bind host receptors. Microbial activity can vary even within the respective compartments of the gut, as these are substantially different environments and therefore total abundance, and compositional nature can differ. We sought to verify that our assay is compatible with different types of gut lumen content, and intestinal material was collected from the small intestine, cecum, and large intestine tissues isolated from mice.

**Fig. 3:**
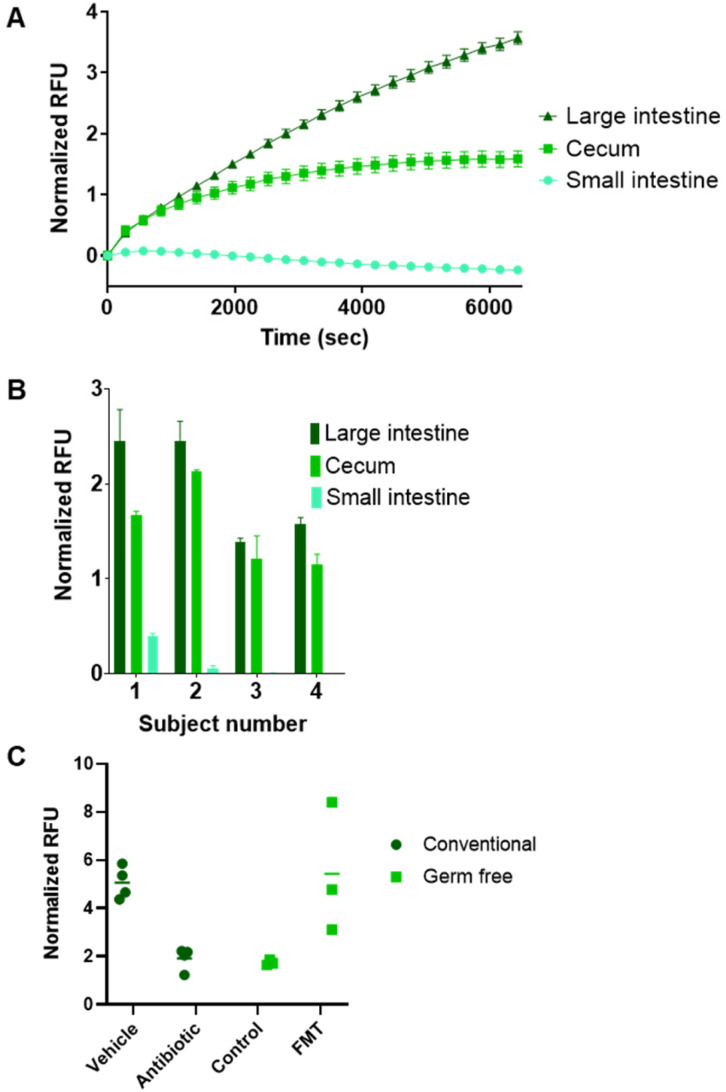
BSH activity varies among compartments of the GI tract. (A) Incubation of ChoRhoS with luminal content collected from the large intestine, cecum, and small intestine from a single C57BL/6J conventional (specific pathogen-free) mouse showed different kinetic profiles for substrate turnover. (B) Examination of BSH activity in each gut compartment of conventional mice for different biological replicates (*n* = 4). (C) C57BL/6J conventional mice treated with an antibiotic cocktail (*n* = 4) showed a significant decrease in fluorescence compared with the mice treated with PBS (*n* = 4) upon co-incubation of the probe with luminal content suspensions (dark green circles). In comparison with C57BL/6J conventional mice treated with vehicle control, large intestine contents from C57BL/6J GF mice (*n* = 3) did not show substantial activity. When C57BL/6J GF mice were inoculated with fecal material (*n* = 3) from C57BL/6J conventional mice *via* oral gavage (FMT, fecal microbiota transplantation), activity increased to a level comparable with conventional mouse controls. All mouse experiments were performed under 12 h/12 h light/dark cycles and with *ad libitum* feeding. Gut compartment study (panel B) was performed at Washington State University's Integrative Physiology and Neuroscience Behavioral Core; antibiotic and FMT studies (panel C) were conducted at Pacific Northwest National Laboratory's Animal Resource Center.

Our assay provided robust kinetic curves for material isolated from the cecum (*p* = 0.0012) and large intestine (*p* = 0.0008), but we observed no substantial activity in the small intestine within the timeframe of our assay (Fig. 3A). These data are in good agreement with what is known about the distribution of bacteria in general, and the specific distribution of BSH-expressing organism in the gut.^55^ We found that these trends were consistent across an examination of four biological replicates (Fig. 3B and S13, S14†). To demonstrate that the evolution of fluorescence was the result of microbial activity, we tested the luminal content of the large intestine in mice treated with an antibiotic cocktail or vehicle control (Fig. 3C) and observed a substantial drop in activity in samples from the antibiotic-treated mice. Additionally, as with fecal samples, we did not observe substantial activity in luminal content from GF mice, but when GF mice received a fecal microbiota transplantation (FMT) from SPF mice, we observed activity levels that were comparable to the control SPF mice (Fig. 3C).

### Characterizing fluctuations in BSH activity throughout the day

**Fig. 4:**
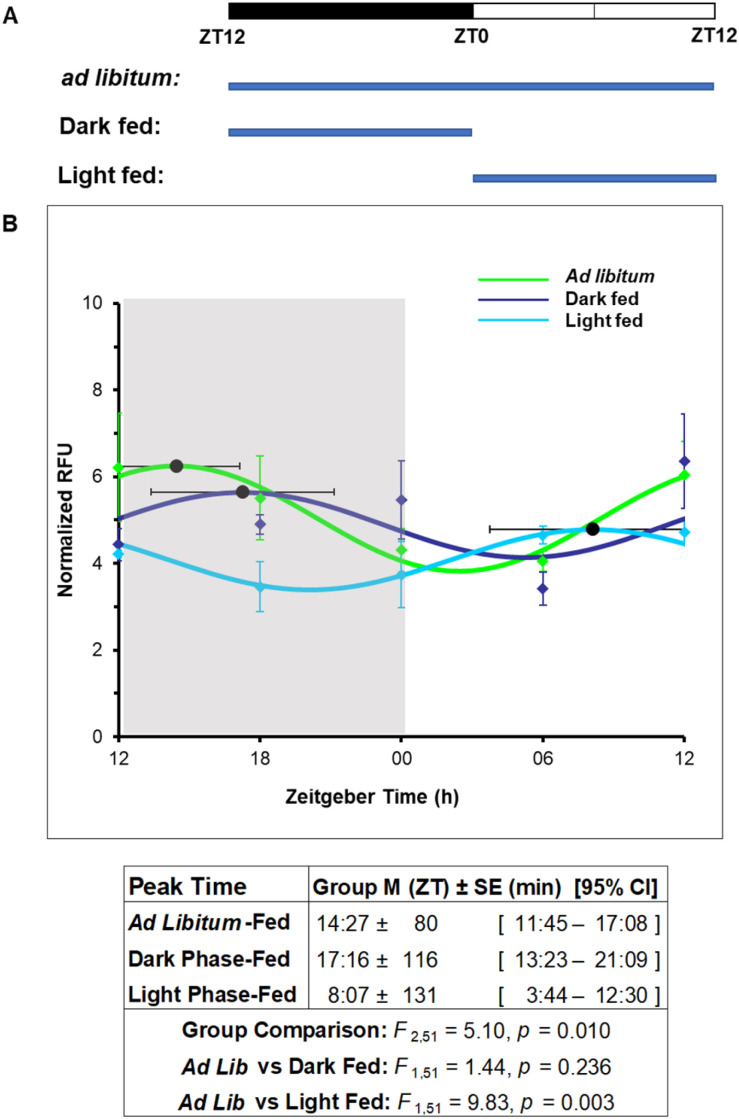
Diurnal rhythms of the BSH activity in gut microbiome are controlled by feeding schedule. (A) Schematics of the feeding protocol. The feeding schedule (*ad libitum*, dark phase-fed, or light phase-fed) was maintained for 2 weeks before sacrificing the mice. ZT (zeitgeber time) represents time of day relative to the start of the light phase (ZT0). (B) Diurnal rhythms of luminal BSH activity in conventional C57BL/6J mice on each of the different feeding schedules (*n* = 4 per time point) showed that fluctuations in the dark phase-fed group were similar to *ad libitum*-fed mice, but the BSH activity rhythm was significantly shifted in the light phase-fed group. Shading indicates dark phase; black horizontal error bars denote 95% CIs of peak times.

Finally, after verifying that our assay is compatible with gut lumen content, we sought to determine if BSH activity is rhythmic in this compartment by analyzing samples acquired from mice on a fixed light/dark schedule. We hypothesized that BSH expressing organisms would exist in the highest abundance during the active phase when feeding occurs and bile would be at its highest concentration in the gut. An experiment was designed in which wild-type SPF mice were maintained on a standard 12 h/12 h light/dark cycle. In one condition of the experiment, the mice had access to food *ad libitum*. Mice are nocturnal animals and are therefore more active and consume more food during the dark phase. We thus expected that we would see the greatest activity with our assay during the dark phase. Mice were sacrificed, in groups of four, at five different time points at 6 h intervals over the course of 24 h, starting at the beginning of the dark phase. Gut lumen content was isolated, and BSH activity was assessed using ChoRhoS. Although there was high variability between biological replicates, the collective data demonstrated rhythmicity in BSH function based on cosinor analysis^56^ (*i.e.*, nonlinear regression analysis fitting a cosine function to activity values as a function of time) (Fig. 4-green and S15–S19†). Our analysis revealed that BSH activity was highest in the middle of the dark phase when the mice are most active and also eat the most. We further hypothesized that, if this rhythmic pattern was regulated by feeding time, a shift in BSH activity would be observed if the feeding time was restricted to a time when the mice are quiescent and more frequently asleep (light phase), and that we should consequently observe the highest BSH activity being shifted to the phase when food was made available. The experiment, therefore, also included a condition in which the 12 h/12 h light/dark cycle was identical to the first experiment, but mice were only provided chow during the light phase when their activity is typically the lowest, and they sleep the most. To account for any effects due to caloric restriction relative to *ad libitum*-fed mice, we also had a control condition in which animals were only offered chow during the dark phase. Similar to the *ad libitum*-fed condition, there was considerable variability between biological replicates for each timepoint, but cosinor analysis demonstrated significantly different rhythms between conditions. In particular, the timing of food availability significantly impacted the timing of the peak in BSH activity (*F*_2,51_ = 5.10, *p* = 0.010). The peak in BSH activity for dark phase-fed mice was about 1.5 h later during the dark phase than that for *ad libitum*-fed mice, a non-significant difference (*p* = 0.236). However, the peak in BSH activity for light phase-fed mice was found during the light phase and more than 6 h earlier than that for *ad libitum*-fed mice, which constituted a significant difference (*p* = 0.003). Thus, there was a difference of almost 9 h in the peak time of BSH activity between the light phase- and dark phase-fed mice.

The peak in BSH activity observed in *ad libitum* and dark phase-fed groups reflects the normal nocturnal behavior of mice. We speculate that the modestly delayed peak for the dark phase-fed mice relative to the *ad libitum*-fed mice may be due to the polyphasic nature of rodent sleep, and that *ad libitum*-fed mice have the opportunity to begin feeding prior to initiation of the dark phase. Conversely, the light phase-fed mice had a BSH activity peak that was much advanced compared to both the dark phase-fed and *ad libitum*-fed mice, which is consistent with our hypothesis regarding the role of feeding time in the regulation of the functional rhythmicity of BSH. Although other methods have established differences in bile salt concentrations throughout the day, these data constitute the first direct demonstration of diurnal rhythmicity in BSH activity, and this is the first study to directly show that BSH activity levels in the large intestine are strongly influenced by feeding patterns.

## Discussion

Bile acid metabolism by the gut microbiome has a substantial influence on human health. Collective BA metabolism in the gut is initiated by BSH, which acts as a crucial player in the coordination of host–microbiome interactions. Development of simple and reliable strategies to detect BSH activity is therefore highly desirable. To specifically characterize BSH as a potential functional oscillator, we developed a simple fluorescence-based tool, ChoRhoS, to directly measure BSH activity rapidly and with high sensitivity. We were able to successfully apply ChoRhoS to the quantification of BSH activity in samples of pure enzyme, microbial monocultures, mixtures of monocultures, and murine feces.

We also applied our ChoRhoS assay to directly monitor BSH activity in samples from the mouse gut lumen. Consistent with what is known about the distribution of BSH-expressing organism in the gut, we found the highest BSH activity in the large intestine and cecum, and negligible activity in the small intestine. We also investigated the impact of the daily light/dark cycle and found that *ad libitum*-fed mice have the highest BSH activity in the large intestine in the dark phase, when the animals are the most active and consume the greatest amounts of food. In addition, we found that we were able to manipulate this trend by restricting the times when food is made available. That is, for mice that received food only during the light period, when they would normally be the least active, peak BSH activity shifted to the light phase. This finding is consistent with a separate study that revealed feeding pattern-based changes in microbiome composition,^32^ and add valuable information regarding functional significance of such changes. Our data indicate that feeding time is a key regulator of BSH activity, and our ChoRhoS assay allowed for the first functional, direct demonstration of this.

The observation of diurnal rhythmicity of BSH activity has important implications because it directly impacts host signaling processes through modification of the occupancy of host BA receptors by free BAs. The possibility that the observed rhythmicity in BSH function may be modified by other factors as well merits further investigation. For example, significant differences in BSH activity between healthy individuals and patients with atherosclerosis and diabetes have been observed.^57^ Moreover, an increase in BSH activity has been reported in a mouse model of colitis compared with healthy mice.^58^ It would be worthwhile to document whether and how these diseases impact the diurnal rhythmicity of BSH activity, and whether alterations in the timing of food intake may have therapeutic potential in this context.

## Conclusions

In conclusion, we developed a simple and efficient tool to detect and quantify BSH enzyme activity in complex biological samples. As microbial BSH enzyme is involved in various host functions such as metabolism and immune function, our strategy can be used to assess host physiology and, eventually, to identify and treat BSH-based functional changes and associated diseases. By using our ChoRhoS tool, accurate estimation of BSH activity can be accomplished, with potential for high throughput applications, which will be particularly beneficial for screening of pharmacological modulators or chemical genetic tools for regulating BSH.

From a broader perspective, based on our observations with BSH, we believe that a function-focused analysis of diurnal rhythmicity of the gut microbiome in general is needed. As the microbiome displays functional rhythmicity this likely impacts rhythmic processes in the host. Previously observed rapid reversal of metabolite rhythmicity,^58,59^ exosome-based metabolic signaling,^60^ and immune parameters^60^ in humans exposed to simulated night shift work may be mediated by functional microbiome rhythm changes. Desynchronization of host-specific circadian processes between tissues leads to development of a variety of diseases,^4^ and it is possible that gut microbiome functions play a major role in circadian desynchronization.^61^ With approximately 20% of the US workforce being exposed to shift work at least some of the time,^3,62^ mitigation of metabolic disturbances caused by circadian desynchronization associated with such work schedules could significantly reduce the societal health burden. As such, it is crucial that we understand which functions of the microbiome are rhythmic, how these rhythms interact with the host, and which are subject to manipulation, so that we can develop robust intervention strategies. We foresee that function-focused assays, such as our ChoRhoS approach, will be key to driving future discoveries.

## Materials and methods

See the ESI† for all the experiment details. All experiments were performed in compliance with the relevant laws and institutional guidelines. All experiments with conventional mice, except for the antibiotic treatment experiment, were conducted at WSU and approved by the WSU IACUC. The experiment conducted with conventional mice involving antibiotic treatment and all experiments conducted with germ-free mice were conducted at PNNL and approved by the PNNL IACUC. Informed consent was obtained for experiments that analyzed human samples.

## Abbreviations

BSH: Bile salt hydrolase
BA: Bile acid
ChoRhoS: CholoylRhodamine substrate
BME: Beta-mercaptoethanol
CA-AMCA: Cholic acid-conjugated amino coumarin probe
CAPE: Caffeic acid phenethyl ester
DMF: Dimethylformamide
FMT: Fecal microbiota transplantation
FS: Fecal suspension
GF: Germ free
HS: Heat-shocked
IACUC: Institutional Animal Care and Use Committee
RFU: Relative fluorescence units
SPF: Specific pathogen-free
ZT: Zeitgeber time

## Author contributions

CJK: conceptualization, methodology, formal analysis, investigation, writing – original draft, writing review & editing, visualization; NA: methodology, investigation; AS: investigation; INK: conceptualization, writing review & editing, HPAVD: conceptualization, formal analysis, writing review & editing, visualization; KRB: conceptualization, methodology, formal analysis, investigation, writing – original draft, writing review & editing, visualization, supervision, project administration, funding acquisition; CJK, NA, IK, HVD, and KRB designed the experiments. CJK, NA, and AS conducted the experiments. CJK, NA, IK, HVD, and KRB analyzed the data. CJK and KRB wrote the manuscript. All authors contributed to revision of the manuscript. All authors have given approval to the final version of the manuscript.

## Conflicts of interest

There are no conflicts to declare.

## Supplementary Material

OB-021-D2OB02257E-s001
