# Supplementary material for: Profiling rhythmicity of bile salt hydrolase activity in the gut lumen with a rapid fluorescence assay
Source: Org Biomol Chem. 2023 Feb 18;21(19):4028–38. doi: 10.1039/d2ob02257e (PMC10191106; doi:10.1039/d2ob02257e)
Supplement: OB-021-D2OB02257E-s001 [file OB-021-D2OB02257E-s001.pdf]

Electronic Supplementary Material (ESI) for Organic & Biomolecular Chemistry.
This journal is © The Royal Society of Chemistry 2023

SUPPORTING INFORMATION

# Profiling rhythmicity of bile salt hydrolase activity in the gut lumen with a rapid fluorescence assay

Chathuri J. Kombala[1,2], Neha Agrawal[1], Agne Sveistyte[1], Ilia N. Karatsoreos[3], Hans P.A. Van Dongen[2,4] and Kristoffer R. Brandvold*[1,5]

1) Biological Sciences Division, Pacific Northwest National Laboratory, Richland, WA 99352 (USA)

2) Department of Translational Medicine and Physiology, Elson S. Floyd College of Medicine, Washington State University, Spokane, WA 99202 (USA)

3) Department of Psychological and Brain Sciences, University of Massachusetts, Amherst, MA 01003 (USA)

4) Sleep and Performance Research Center, Washington State University, Spokane, WA 99202 (USA)

5) Department of Nutrition and Exercise Physiology, Elson S. Floyd College of Medicine, Washington State University, Spokane, WA 99202 (USA)

Email: kristoffer.brandvold@pnnl.gov

## TABLE OF CONTENTS


| | | |
|---|---|---|
| I. | Supplementary data | S3-S20 |
| II. | Experimental procedures | S21-S23 |
| | a. General synthetic methods | |
| | b. Synthetic procedures | |
| III. | Biological characterization of probes | S24-S26 |
| IV. | Animal experiments and sample collection | S27-28 |
| V. | Procedures for detection of bacterial contamination in gnotobiotic mice | S29 |
| VI. | Statistical analyses | S30 |
| VII. | Spectral data | S31-36 |
| VIII. | References | S37 |

# I. SUPPLEMENTARY DATA

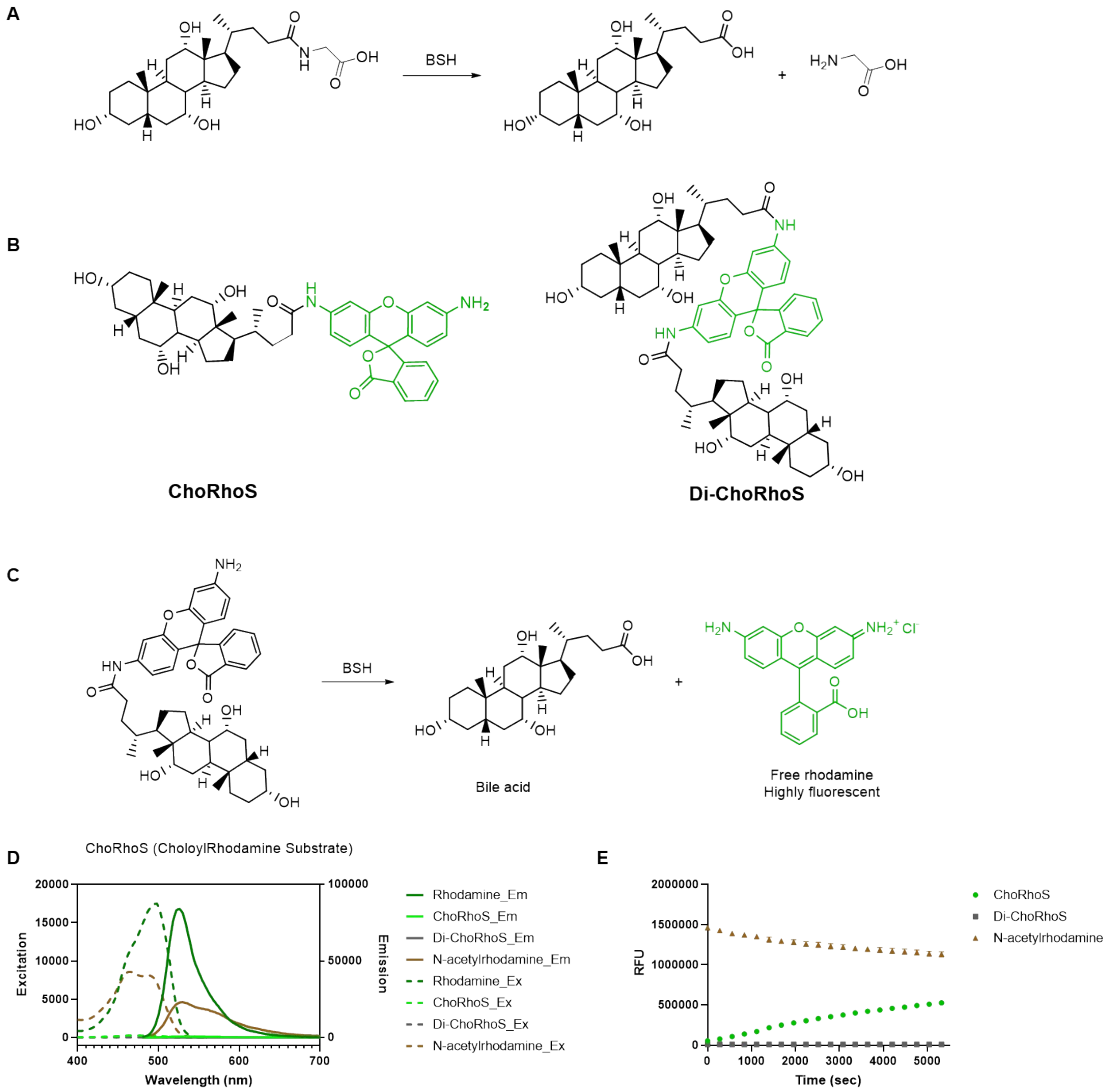


**Figure S1. Biochemical and spectral characterization of the probe.** A) Bile salt hydrolase (BSH) is a key regulator of bile acid homeostasis. Conjugated bile acids (BA) are metabolized into deconjugated BAs by the BSH enzyme in the gut microbiota. B) Structures of ChoRhoS and Di-ChoRhoS substrates. C) Fluorogenic BSH substrate was developed by conjugating rhodamine to cholic acid. Upon exposure of synthetic substrate to BSH, the amide bond is hydrolyzed and produces a free BA and free rhodamine, which is a highly fluorogenic compound compared with its conjugated version. D) Excitation and emission spectra of free rhodamine, ChoRhoS, Di-ChoRhoS and N-acetyl rhodamine. Excitation and emission spectra of ChoRhoS and Di-ChoRhoS are overlapped and difficult to distinguish due to their low fluorescence). E) Rhodamine

fluorescence is significantly increased in the presence of BSH only. The probe fluorescence does not change in buffer.

Table S1: Spectroscopic properties of ChoRhoS acquired at 25 ˚C in 1x PBS, pH 7.3.

| | $\lambda_{ex}$ (nm) | $\lambda_{em}$ (nm) | $\varepsilon$( $M^{-1}cm^{-1}$) |
|---|---|---|---|
| *ChoRhoS* | 495 | 525 | 1724.13 |

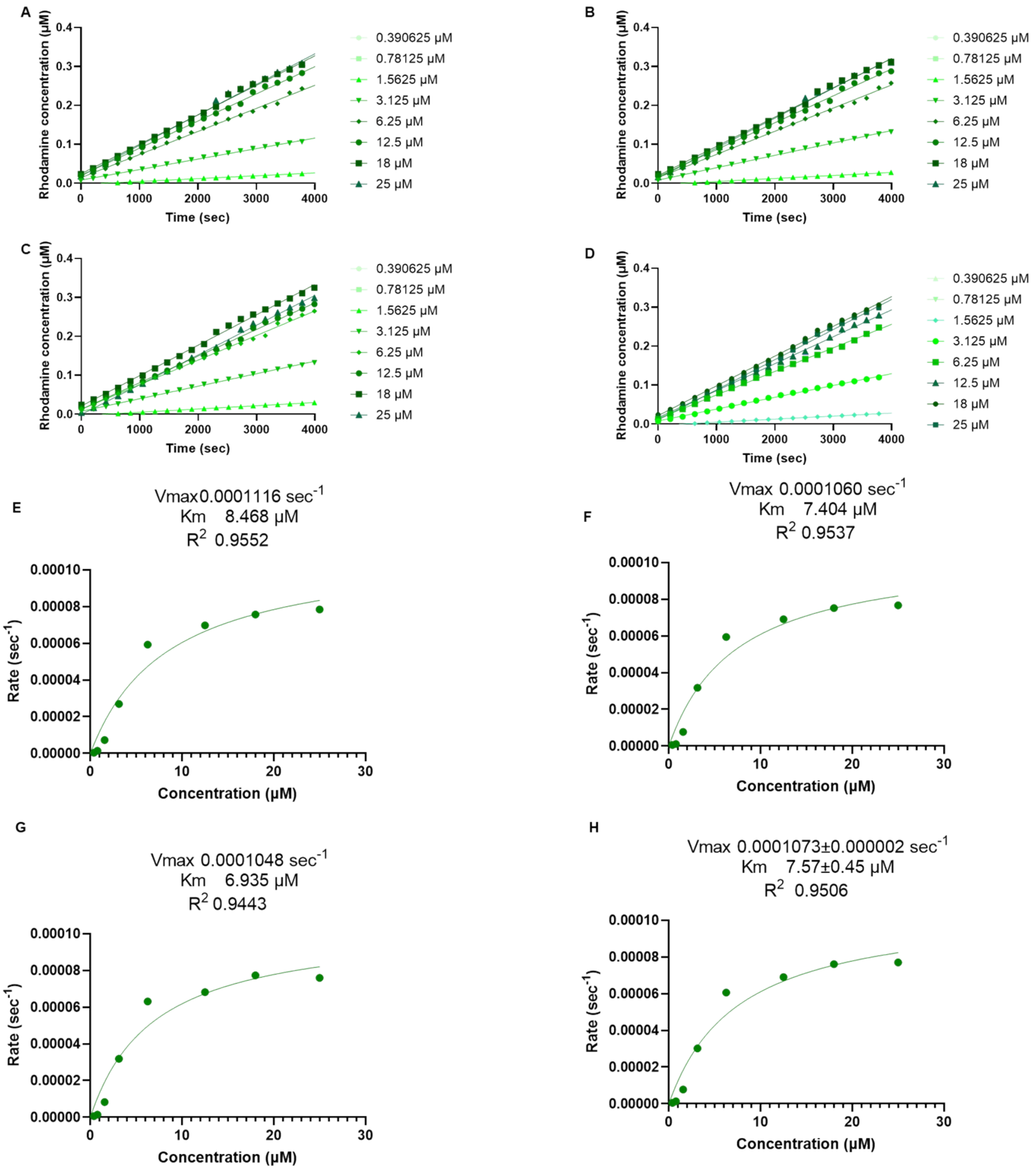


**Figure S2. $K_M$ determination for ChoRhoS probe**. Buffered solutions of 40 µg/mL purified BSH were treated with varying concentrations of substrate, and slopes of the initial linear phase were used to determine the rate of the reaction at each substrate concentration. The kinetic properties of substrate hydrolysis by BSH were determined using a Michaelis-Menten (MM) analysis. A, B, C are substrate concentration kinetic plots for three replicates and D is the average plot for all. E, F, G are rate curves for each replicate and H is the average rate plot for all 3 replicates.

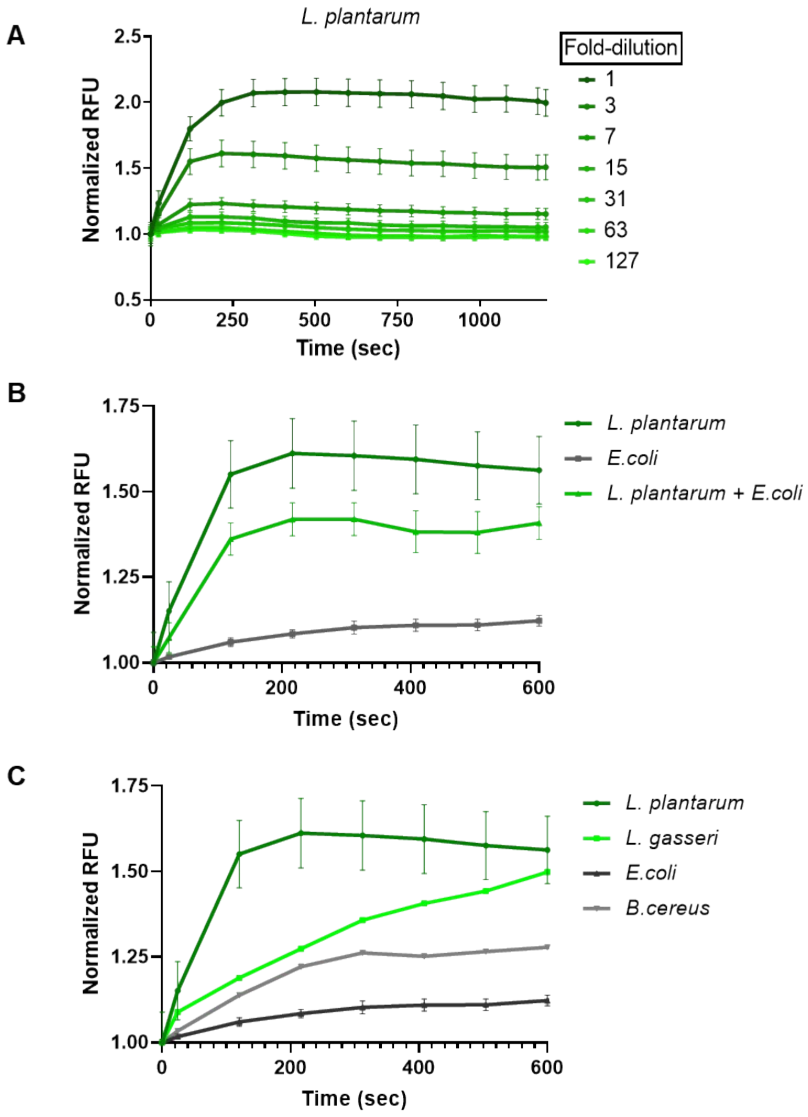


**Figure S3. ChoRhoS fluorogenic substrate is only cleaved with whole-cell monocultures that express BSH.** *L. plantarum* and RFP (Red Fluorescent Protein) *E. coli* cells were cultured in MRS (De Man, Rogosa and Sharpe agar) and LB (Lysogeny Broth) media, respectively, until optical density reached 0.6. The final cell pellet was suspended in 1000 µL buffer and serially diluted to obtain different cell densities. A) Incubation of *L. plantarum* monoculture with ChoRhoS showed an increase in fluorescence over time at different cell densities. B) Comparison of *L. plantarum* and *E. coli* mono-cultures at the same cell density showed significantly lower fluorescence ($p<0.0001$) for *E. coli.* A 1:1 mixture of *E. coli* and *L. plantarum* cells showed an increase in fluorescence over time. The lower fluorescence observed in mixture could be due to the half-cell density of *L. plantarum* and *E. coli* cells compared with mono-cultures. C) Significant probe cleavage was observed in BSH expressing mono-cultures (*L. plantarum* and *L. gasseri),* but not in non-BSH expressing mono-cultures (*E. coli* and *B. cereus*), at the same cell density. ChoRhoS (25 µM) was co-incubated with cell suspensions at 37 °C, and probe hydrolysis was quantified by measuring fluorescence at 495 nm and 525 nm excitation and emission wavelengths, respectively, using a plate reader.

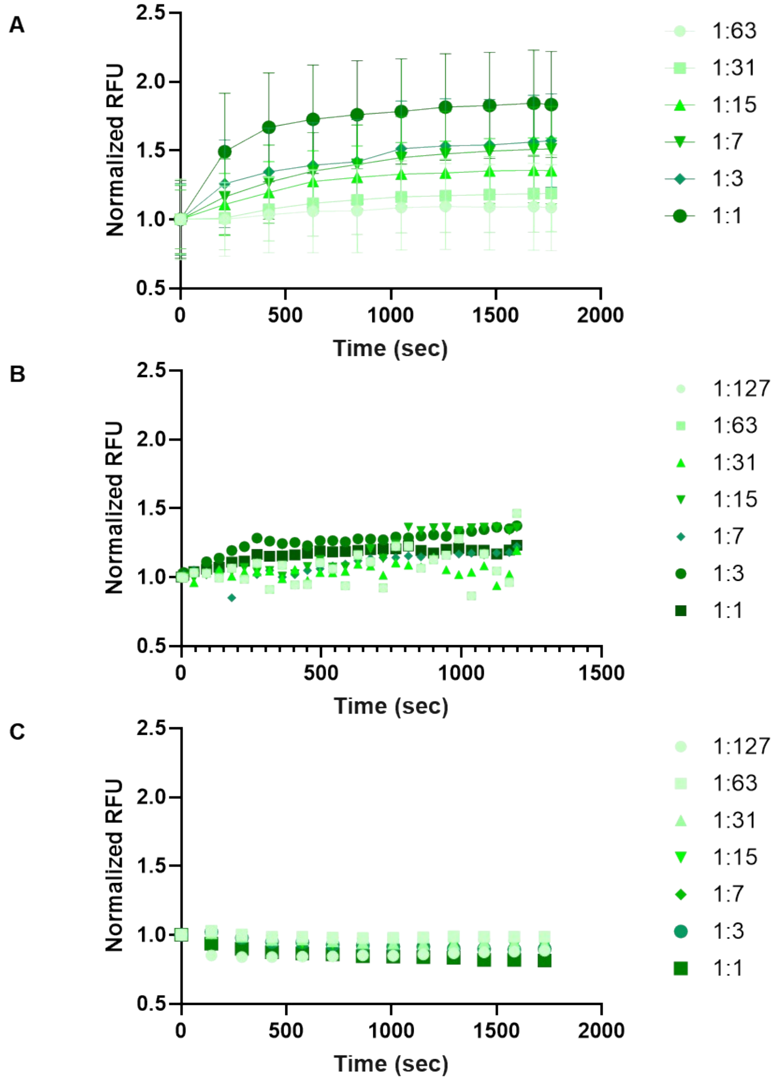


**Figure S4. ChoRhoS fluorogenic substrate is only turned over in cell lines that have BSH.** RFU plots for different cell lines are shown. Addition of ChoRhoS (50 μM) to whole cell suspensions of A) *L. gasseri* showed increase in fluorescence, whereas B) *B. cereus* and C) *E. coli* Nissle, which do not express BSH, did not cause an increase in fluorescence**.**

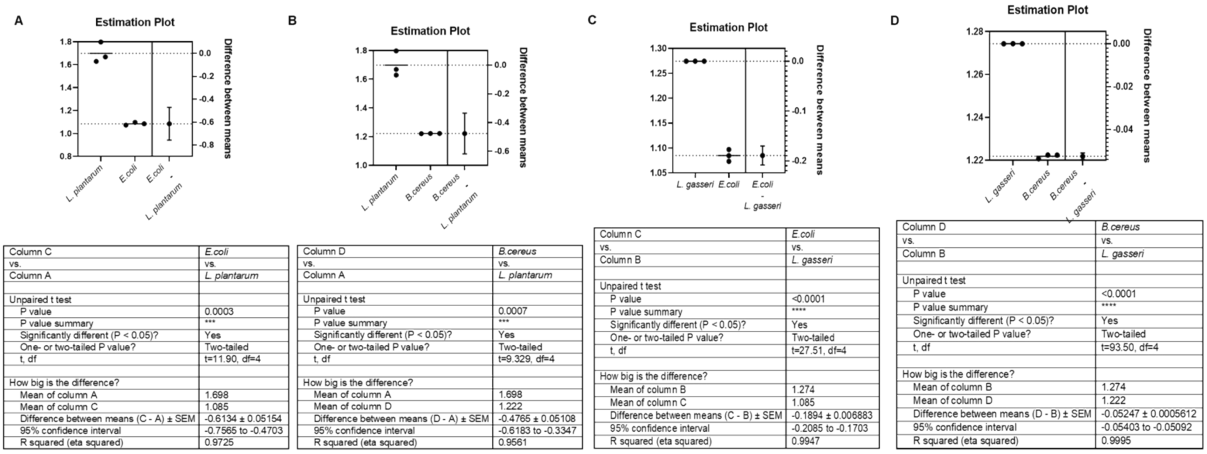


| | |
|---|---|
| Column C | *E.coli* |
| vs. | vs. |
| Column A | *L. plantarum* |
| | |
| Unpaired t test | |
| P value | 0.0003 |
| P value summary | *** |
| Significantly different (P < 0.05)? | Yes |
| One- or two-tailed P value? | Two-tailed |
| t, df | t=11.90, df=4 |
| | |
| How big is the difference? | |
| Mean of column A | 1.698 |
| Mean of column C | 1.085 |
| Difference between means (C - A) ± SEM | -0.6134 ± 0.05154 |
| 95% confidence interval | -0.7565 to -0.4703 |
| R squared (eta squared) | 0.9725 |

| | |
|---|---|
| Column D | *B.cereus* |
| vs. | vs. |
| Column A | *L. plantarum* |
| | |
| Unpaired t test | |
| P value | 0.0007 |
| P value summary | *** |
| Significantly different (P < 0.05)? | Yes |
| One- or two-tailed P value? | Two-tailed |
| t, df | t=9.329, df=4 |
| | |
| How big is the difference? | |
| Mean of column A | 1.698 |
| Mean of column D | 1.222 |
| Difference between means (D - A) ± SEM | -0.4765 ± 0.05108 |
| 95% confidence interval | -0.6183 to -0.3347 |
| R squared (eta squared) | 0.9561 |

| | |
|---|---|
| Column C | *E.coli* |
| vs. | vs. |
| Column B | *L. gasseri* |
| | |
| Unpaired t test | |
| P value | <0.0001 |
| P value summary | **** |
| Significantly different (P < 0.05)? | Yes |
| One- or two-tailed P value? | Two-tailed |
| t, df | t=27.51, df=4 |
| | |
| How big is the difference? | |
| Mean of column B | 1.274 |
| Mean of column C | 1.085 |
| Difference between means (C - B) ± SEM | -0.1894 ± 0.006883 |
| 95% confidence interval | -0.2085 to -0.1703 |
| R squared (eta squared) | 0.9947 |

| | |
|---|---|
| Column D | *B.cereus* |
| vs. | vs. |
| Column B | *L. gasseri* |
| | |
| Unpaired t test | |
| P value | <0.0001 |
| P value summary | **** |
| Significantly different (P < 0.05)? | Yes |
| One- or two-tailed P value? | Two-tailed |
| t, df | t=93.50, df=4 |
| | |
| How big is the difference? | |
| Mean of column B | 1.274 |
| Mean of column D | 1.222 |
| Difference between means (D - B) ± SEM | -0.05247 ± 0.0005612 |
| 95% confidence interval | -0.05403 to -0.05092 |
| R squared (eta squared) | 0.9995 |

**Figure S5. Statistical analysis of fluorescence evolution comparing BSH expressing and non-BSH expressing cell lines.** Unpaired t-test results are shown for comparisons of A) *L. plantarum* and *E. coli,* B) *L. plantarum* and *B. cereus,* C) *L. gasseri* and *E. coli,* and D) *L. gasseri* and *B. cereus.*

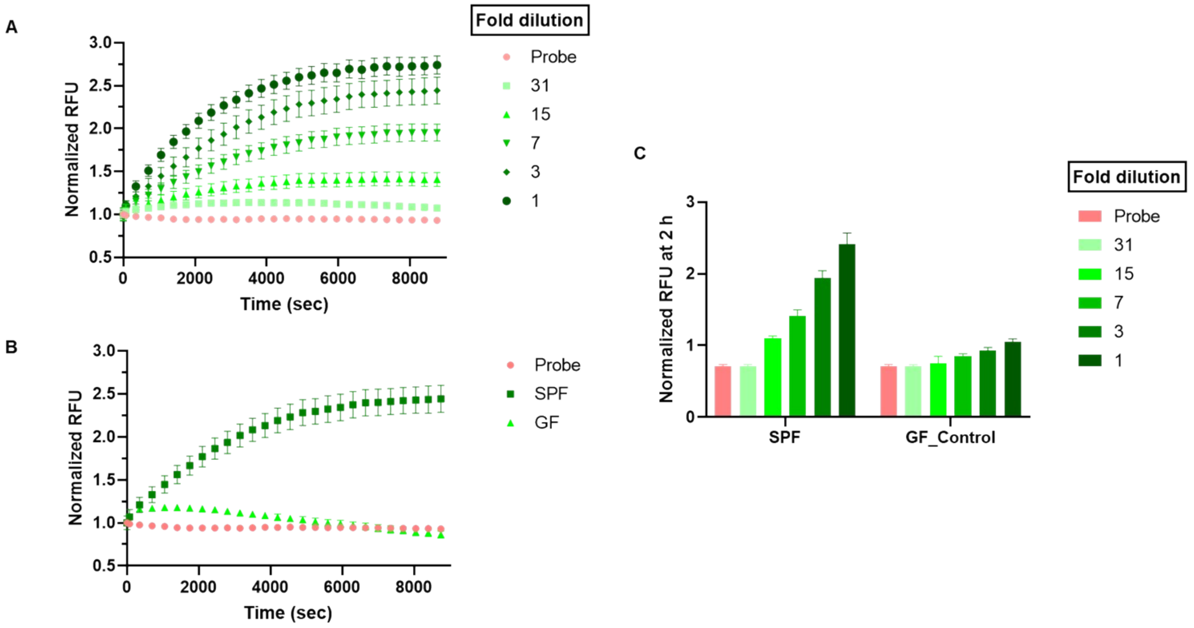


**Figure S6. RFU plots for fecal sample characterization with ChoRhoS probe.** A) SPF mice with different fecal suspension dilutions, B) GF and SPF fecal suspension comparison, and C) RFU plot at 6000 s for different dilutions.

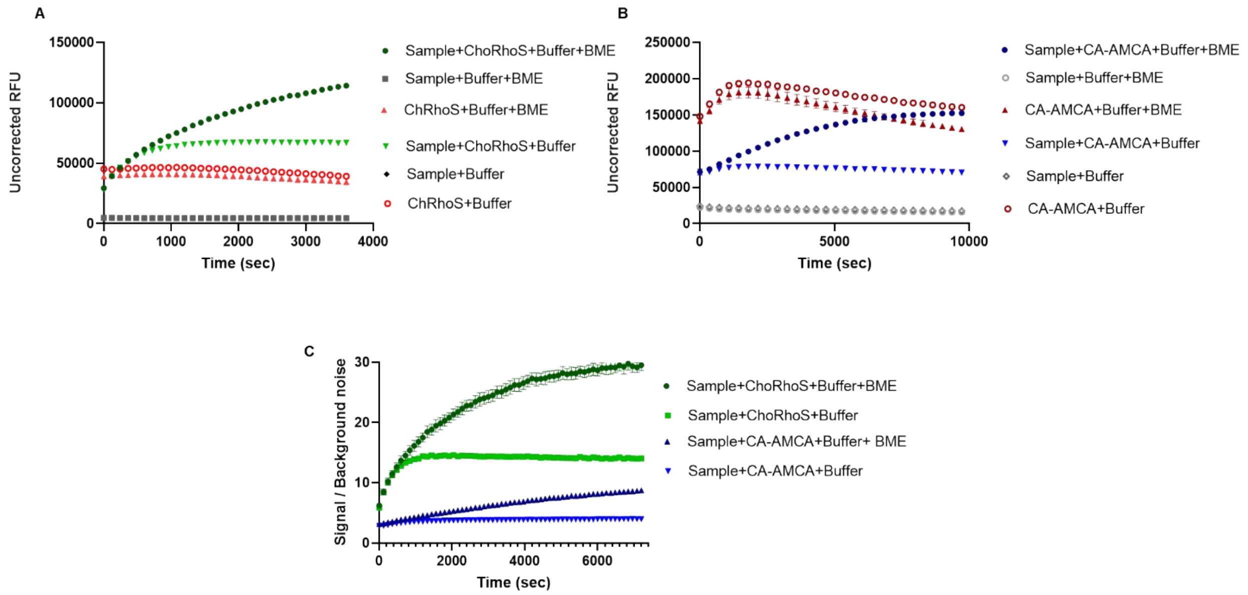


**Figure S7. ChoRhoS probe fluorescence is higher than CA-AMCA probe fluorescence when incubated with mouse fecal suspension.** Two fecal samples (50 mg) were suspended in 1 mL of 1x PBS with and without beta-mercaptoethanol (BME) and incubated with both ChoRhoS and CA-AMCA probes at 37 °C. The reaction fluorescence, background fluorescence signal from the fecal suspensions, and probe blank fluorescence were quantified by measuring fluorescence at 495 nm and 525 nm for ChoRhoS and 350 nm and 450 nm for CA-AMCA, using a plate reader. A) ChoRhoS probe is cleaved in the presence and absence of BME. However, the probe turnover is much higher in the presence of BME. B) CA-AMCA probe is cleaved in the presence and absence of BME. However, background signal is significantly higher, and fluorescence turn-over is lower than probe blank. C) The S/N ratio is 3-fold higher for ChoRhoS than for CA-AMCA after 2 h. Sample+buffer and Sample+buffer+BME curves in figure A and B showed very low fluorescence and RFU plots overlapped on each other.

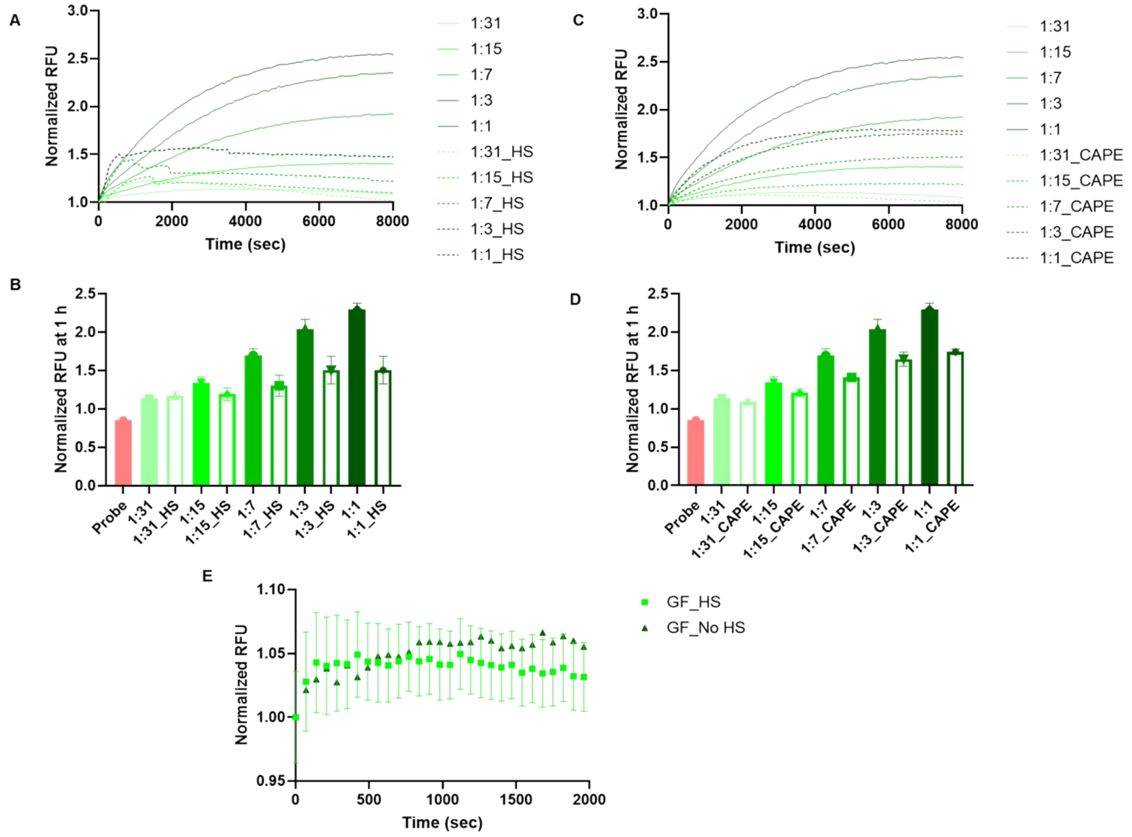


**Figure S8. Pre-treatment of fecal suspensions with heat-shock (HS) or BSH inhibitor lowers BSH activity in fecal samples.** Panels show fluorescence intensity of A, B) heat-shocked (HS) SPF fecal samples with non-HS controls and C, D) fecal samples treated with a BSH inhibitor, caffeic acid phenethyl ester (CAPE) with no CAPE controls, at different dilutions. E) There was no significant difference in fluorescence between heat-shocked GF samples and non-heat-shocked samples, implying that minor fluorescence increase observed in GF samples is not from microbial activity. Error bars in A and C panels omitted for clarity.

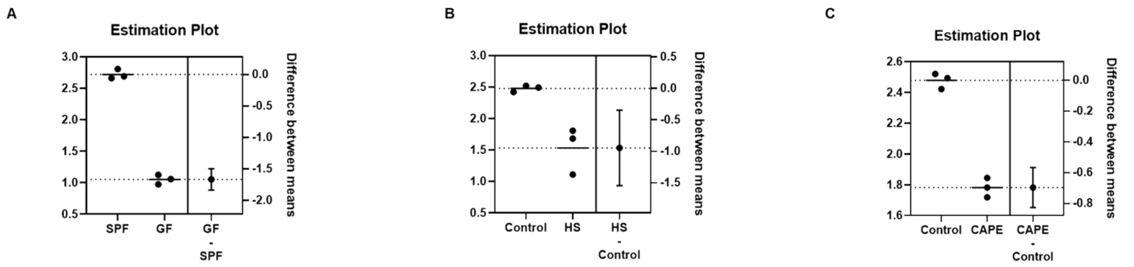


| | |
|---|---|
| Column B | GF |
| vs. | vs. |
| Column A | SPF |
| | |
| Unpaired t test | |
| P value | <0.0001 |
| P value summary | **** |
| Significantly different (P < 0.05)? | Yes |
| One- or two-tailed P value? | Two-tailed |
| t, df | t=27.17, df=4 |
| | |
| How big is the difference? | |
| Mean of column A | 2.719 |
| Mean of column B | 1.052 |
| Difference between means (B - A) ± SEM | -1.667 ± 0.06135 |
| 95% confidence interval | -1.837 to -1.497 |
| R squared (eta squared) | 0.9946 |

| | |
|---|---|
| Column B | HS |
| vs. | vs. |
| Column A | Control |
| | |
| Unpaired t test | |
| P value | 0.0119 |
| P value summary | * |
| Significantly different (P < 0.05)? | Yes |
| One- or two-tailed P value? | Two-tailed |
| t, df | t=4.381, df=4 |
| | |
| How big is the difference? | |
| Mean of column A | 2.479 |
| Mean of column B | 1.532 |
| Difference between means (B - A) ± SEM | -0.9465 ± 0.2161 |
| 95% confidence interval | -1.546 to -0.3466 |
| R squared (eta squared) | 0.8275 |

| | |
|---|---|
| Column C | CAPE |
| vs. | vs. |
| Column A | Control |
| | |
| Unpaired t test | |
| P value | 0.0001 |
| P value summary | *** |
| Significantly different (P < 0.05)? | Yes |
| One- or two-tailed P value? | Two-tailed |
| t, df | t=14.91, df=4 |
| | |
| How big is the difference? | |
| Mean of column A | 2.479 |
| Mean of column C | 1.782 |
| Difference between means (C - A) ± SEM | -0.6967 ± 0.04674 |
| 95% confidence interval | -0.8265 to -0.5670 |
| R squared (eta squared) | 0.9823 |

**Figure S9. Statistical analysis for comparison of fecal suspensions with and without gut microbiome, and comparison of pre-heat-shocked and pre-BSH inhibitor treated fecal suspensions with non-treated fecal suspensions.** A) Unpaired t-test for comparison of normalized fluorescence evolution from GF and SPF mice fecal suspension at 2h time point. B) Unpaired t-test for comparison of fluorescence evolution in non-heat-shocked and heat-shocked fecal samples (n=3 per group). C) Unpaired t-test for comparison of fluorescence evolution in fecal samples without and with addition of BSH inhibitor (CAPE) (n=3 per group).

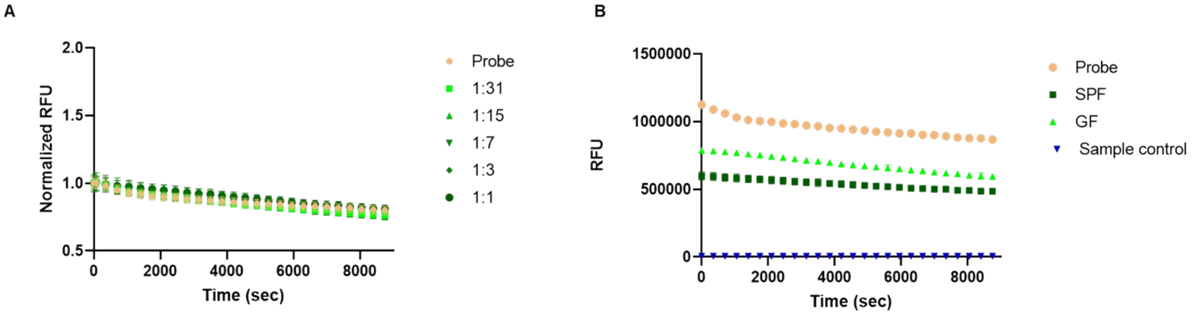


**Figure S10. BSH specifically cleaves the cholic acid conjugated rhodamine substrate.** A) Incubation of different dilutions of SPF mice fecal suspensions with N-acetylrhodamine probe did not show increases in fluorescence. B) Upon incubation with N-acetylrhodamine, neither GF nor SPF fecal suspensions showed an increase in fluorescence.

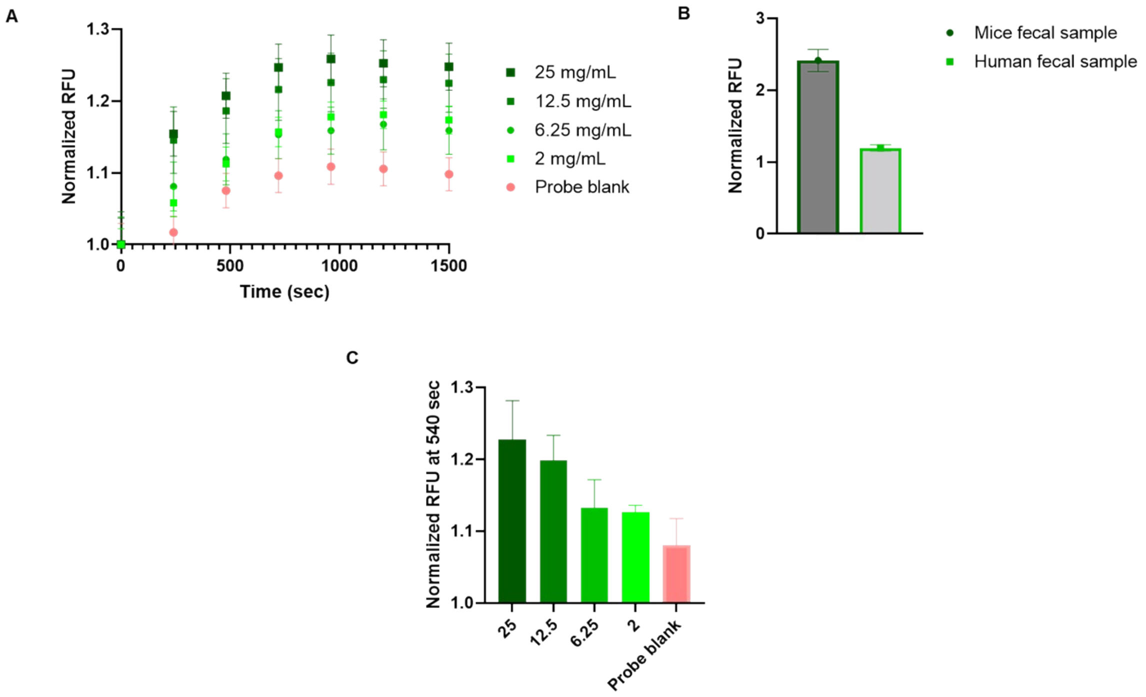


**Figure S11. Preliminary data for human fecal sample analysis.** A) Kinetic profiles for different dilutions of single human fecal sample. RFU values were normalized by dividing by t=0 value (first RFU read). B) Comparison of fold change over t=0 value for mice and human fecal samples. C) Normalized RFU profiles for different dilutions of fecal sample and probe blank at 540 s. Normalized RFU values are significantly different from probe blank at higher concentrations of fecal slurries (12.5 mg/L: p=0.016; 25 mg/mL: p=0.023).

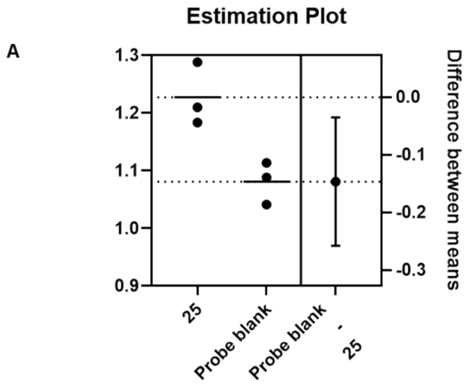


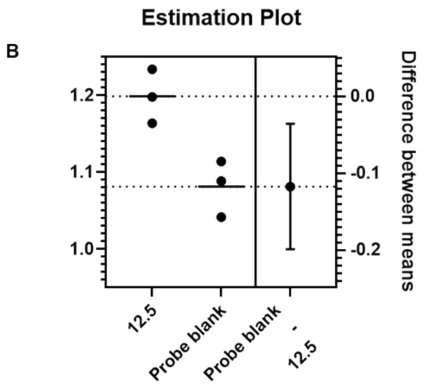


| | |
|---|---|
| Column E | Probe blank |
| vs. | vs. |
| Column A | 25 |
| | |
| Unpaired t test with Welch's correction | |
| P value | 0.0232 |
| P value summary | * |
| Significantly different (P < 0.05)? | Yes |
| One- or two-tailed P value? | Two-tailed |
| Welch-corrected t, df | t=3.857, df=3.508 |
| | |
| How big is the difference? | |
| Mean of column A | 1.227 |
| Mean of column E | 1.081 |
| Difference between means (E - A) ± SEM | -0.1462 ± 0.03791 |
| 95% confidence interval | -0.2576 to -0.03488 |
| R squared (eta squared) | 0.8092 |

| | |
|---|---|
| Column E | Probe blank |
| vs. | vs. |
| Column B | 12.5 |
| | |
| Unpaired t test with Welch's correction | |
| P value | 0.0162 |
| P value summary | * |
| Significantly different (P < 0.05)? | Yes |
| One- or two-tailed P value? | Two-tailed |
| Welch-corrected t, df | t=3.997, df=3.992 |
| | |
| How big is the difference? | |
| Mean of column B | 1.198 |
| Mean of column E | 1.081 |
| Difference between means (E - B) ± SEM | -0.1173 ± 0.02935 |
| 95% confidence interval | -0.1988 to -0.03575 |
| R squared (eta squared) | 0.8000 |

**Figure S12. Statistical analysis of fluorescence evolution from human fecal samples compared to probe blank.** Unpaired t-test results are shown for comparisons of probe blank and fecal suspension at A) 25 mg/mL and B) 12.5 mg/mL. The fluorescence evolution from human fecal suspensions at these high concentrations was significantly different from the probe blank.

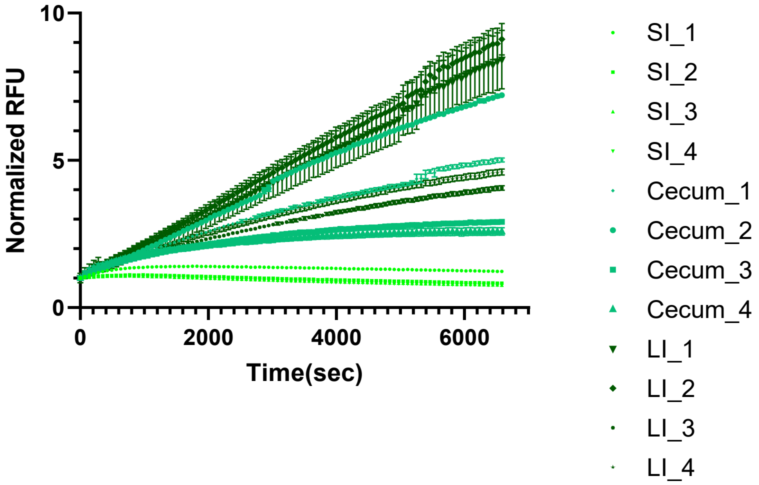


**Figure S13. Kinetic profiles for probe incubated with different luminal content suspensions.** Data are shown for all biological replicates (n=4) from the small intestine (SI), cecum, and large intestine (LI).

A

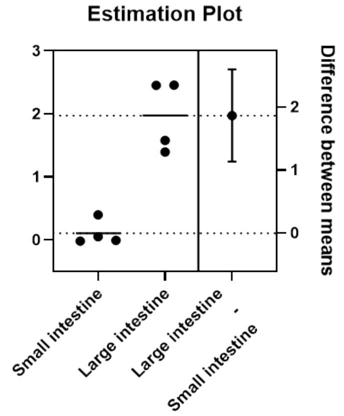


| | |
|---|---|
| Column C | Large intestine |
| vs. | vs. |
| Column A | Small intestine |
| | |
| Unpaired t test | |
| P value | 0.0008 |
| P value summary | *** |
| Significantly different (P < 0.05)? | Yes |
| One- or two-tailed P value? | Two-tailed |
| t, df | t=6.235, df=6 |
| | |
| How big is the difference? | |
| Mean of column A | 0.1052 |
| Mean of column C | 1.971 |
| Difference between means (C - A) ± SEM | 1.865 ± 0.2992 |
| 95% confidence interval | 1.133 to 2.598 |
| R squared (eta squared) | 0.8663 |

B

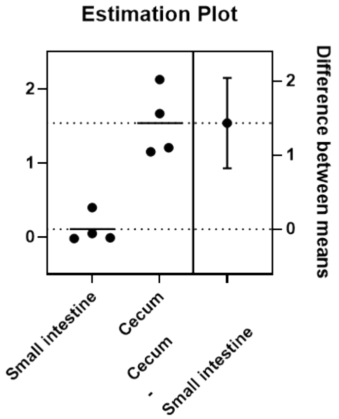


| | |
|---|---|
| Column B | Cecum |
| vs. | vs. |
| Column A | Small intestine |
| | |
| Unpaired t test | |
| P value | 0.0012 |
| P value summary | ** |
| Significantly different (P < 0.05)? | Yes |
| One- or two-tailed P value? | Two-tailed |
| t, df | t=5.769, df=6 |
| | |
| How big is the difference? | |
| Mean of column A | 0.1052 |
| Mean of column B | 1.538 |
| Difference between means (B - A) ± SEM | 1.432 ± 0.2483 |
| 95% confidence interval | 0.8248 to 2.040 |
| R squared (eta squared) | 0.8472 |

**Figure S14. Statistical analysis of fluorescence evolution comparing different compartments of the gut content.** Unpaired t-test results are shown for comparisons of A) small intestine vs. large intestine, and B) small intestine vs. cecum contents (gut contents were isolated from n=4 mice per group, separately, and analyzed separately). When incubated with the probe, fluorescence evolution from large intestine content ($p=0.0008$) and cecum ($p=0.0012$) was significantly higher than from small intestine content.

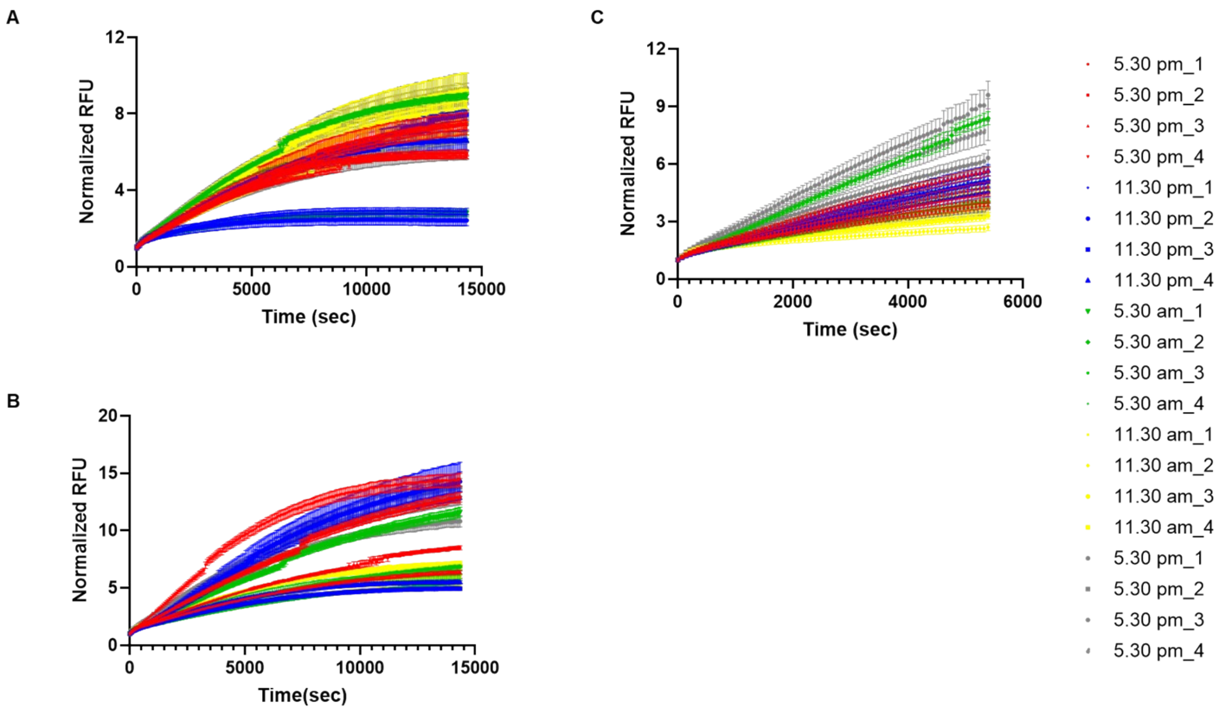


**Figure S15. Substrate turnover kinetic profiles for mice on different feeding schedules.** Data are shown for the individual replicates at different time points of mice (n=4 per time point) in the A) *ad libitum*-fed group, B) light-fed group, and C) dark-fed group; n=20 mice per feeding condition were used for the analysis.

A) B)

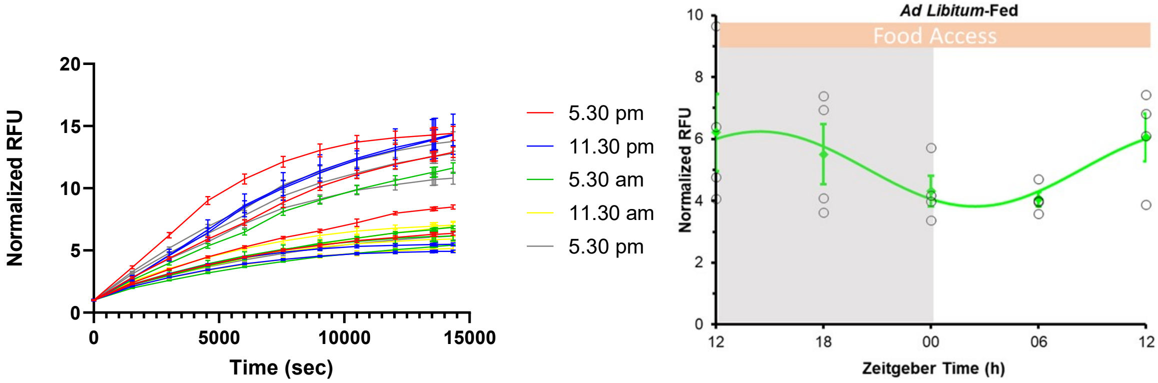


**Figure S16. BSH activity for *ad libitum*-fed mice.** A) Raw RFU velocity data for all biological replicates. B) RFU values for each biological replicate at 5040 s represented as single points (open circles), means and standard errors by time point (solid diamonds and error bars), and cosinor analysis for diurnal rhythmicity (fitted curve).

A) B)

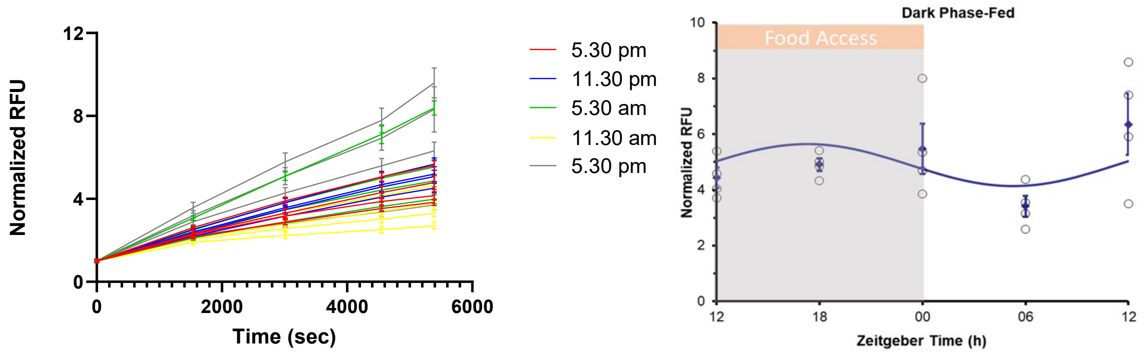


**Figure S17. BSH activity for dark-fed mice.** A) Raw RFU velocity data for all biological replicates. B) RFU values for each biological replicate at 5040 s represented as single points (open circles), means and standard errors by time point (solid diamonds and error bars), and cosinor analysis for diurnal rhythmicity (fitted curve).

**A)**

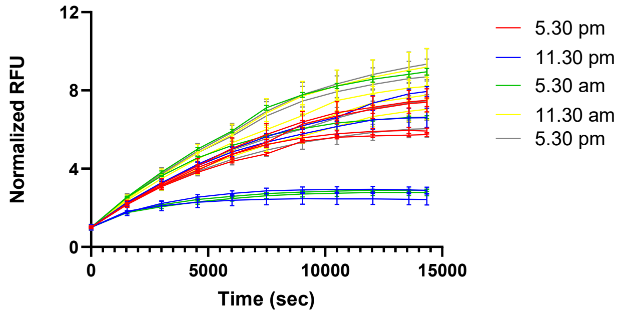


**B)**

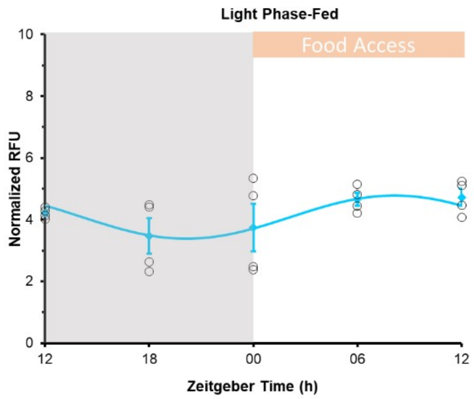


**Figure S18. BSH activity for light-fed mice.** A) Raw RFU velocity data for all biological replicates. B) RFU values for each biological replicate at 5040 s represented as single points (open circles), means and standard errors by time point (solid diamonds and error bars), and cosinor analysis for diurnal rhythmicity (fitted curve).

**A**

| Amplitude | Group M ± SE ($t$ test, $p$ value) |
|---|---|
| *Ad Libitum* -Fed | 1.22 ± 0.41 ($t_{51}$ = 2.99 , $p$ = 0.004 ) |
| Dark Phase-Fed | 0.76 ± 0.45 ($t_{51}$ = 1.69 , $p$ = 0.097 ) |
| Light Phase-Fed | 0.70 ± 0.43 ($t_{51}$ = 1.62 , $p$ = 0.111 ) |
| **Group Comparison:** $F_{2,51}$ = 0.46, $p$ = 0.636 | |

**B**

| Peak Time | Group M (ZT) ± SE (min) [95% CI] |
|---|---|
| *Ad Libitum* -Fed | 14:27 ± 80 [ 11:45 – 17:08 ] |
| Dark Phase-Fed | 17:16 ± 116 [ 13:23 – 21:09 ] |
| Light Phase-Fed | 8:07 ± 131 [ 3:44 – 12:30 ] |
| **Group Comparison:** $F_{2,51}$ = 5.10, $p$ = 0.010 | |

**C**

| Mesor | Group M ± SE |
|---|---|
| *Ad Libitum* -Fed | 5.03 ± 0.29 |
| Dark Phase-Fed | 4.89 ± 0.29 |
| Light Phase-Fed | 4.08 ± 0.29 |
| **Comparison:** $F_{2,51}$ = 2.99, $p$ = 0.059 | |

**Model goodness of fit:** $F_{8,40}$ = 3.21, $p$ = 0.007

**Figure S19. Statistical analysis of diurnal rhythmicity of BSH activity in mice by feeding schedule.** Tables show cosinor analysis results (means ± SE) for A) amplitude (with test of rhythm significance), B) peak time (with 95% confidence interval) relative to onset of light phase (ZT, Zeitgeber time), and C) mesor (mean level), for all three conditions (*Ad libitum*-fed, Dark phase-fed, Light phase-fed), as well as overall comparison between conditions.

## II. EXPERIMENTAL PROCEDURES

### a) GENERAL SYNTHETIC METHODS

Unless otherwise noted, all reagents and solvents were obtained from commercial sources and used without further purification. Chemical shifts are reported in ppm (δ) referenced to the NMR solvent residual peak, and coupling constants (J) are in Hz. All reactions were monitored using TLC and LTQ-MS. For characterization of new compounds, $^{1}H$, $^{13}C$ NMR and LTQ-MS data have been included. Mass Spectrometry (HRMS) was carried out at PNNL (Ronald J. Moore).

### b) SYNTHETIC PROCEDURES

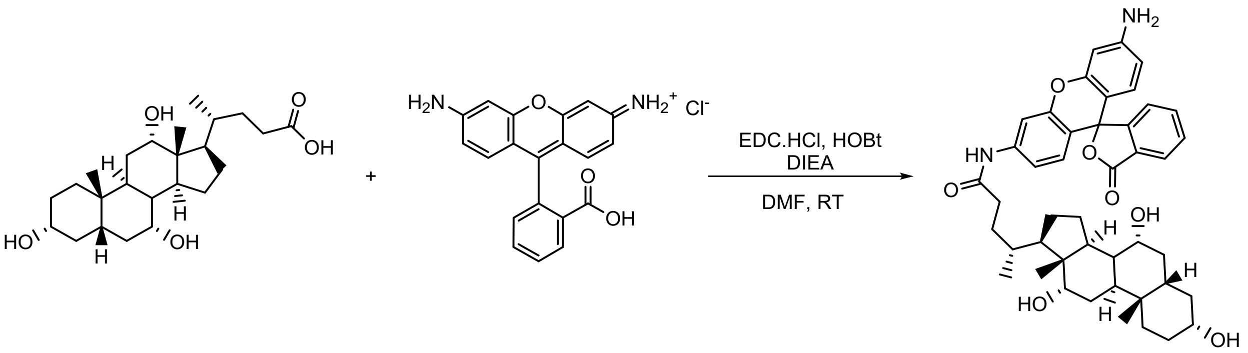


**Scheme S1. Chemical synthesis of ChoRhoS.** Cholic acid (122.2 mg, 0.29 mmol), HOBt (38.67 mg, 0.29 mmol), DIEA (35.2 mg, 0.27 mmol) and rhodamine-110 (100 mg, 0.272 mmol) were dissolved in 3 mL of anhydrous DMF. The reaction mixture was cooled to 0 °C, then EDC.HCl (57.26 mg, 0.29 mmol) was added and allowed to stir for 48 h. The reaction was diluted with ethyl acetate and organic layer was washed with water followed by brine and dried with anhydrous sodium sulfate. The crude product was purified by flash chromatography (0-12%, Methanol:DCM) to obtain the final compound as an orange powder (Yield: 46 mg, 23%).

$^{1}H$ NMR (400 MHz, DMSO-$d_6$) δ 10.14 (s, 1H), 7.99 (dt, $J$ = 7.6, 1.0 Hz, 1H), 7.83 (t, $J$ = 1.8 Hz, 1H), 7.79 (td, $J$ = 7.5, 1.2 Hz, 1H), 7.71 (td, $J$ = 7.5, 1.0 Hz, 1H), 7.26 (d, $J$ = 7.6 Hz, 1H), 7.09 (dt, $J$ = 8.7, 1.7 Hz, 1H), 6.64 (d, $J$ = 8.7 Hz, 1H), 6.45 (d, $J$ = 2.1 Hz, 1H), 6.41 – 6.30 (m, 2H), 5.67 (s, 2H), 4.32 (d, $J$ = 4.3 Hz, 1H), 4.11 (dd, $J$ = 7.9, 4.3 Hz, 1H), 4.02 (d, $J$ = 3.5 Hz, 1H), 3.80 (d, $J$ = 3.5 Hz, 1H), 3.62 (s, 1H), 3.18 (d, $J$ = 5.2 Hz, 1H), 2.30 – 2.11 (m, 2H), 2.10 – 1.94 (m, 1H), 1.92 – 1.60 (m, 8H), 1.53 – 1.11 (m, 10H), 0.98 (d, $J$ = 6.2 Hz, 4H), 0.82 (s, 3H), 0.60 (s, 3H). 13C NMR (101 MHz, DMSO-d6) δ 172.84, 169.27, 152.98, 152.45, 151.83, 151.63, 141.73, 135.97, 130.45, 128.95, 128.69, 126.79, 125.01, 124.48, 115.18, 113.80, 111.70, 106.51, 105.66, 99.60, 83.97, 71.47, 70.90, 66.70, 49.07, 46.59, 46.22, 41.99, 41.86, 35.78, 35.63, 35.36, 34.86, 34.01, 31.82, 30.87, 29.04, 27.76, 26.69, 23.28, 23.10, 17.61, 12.84. HRMS (m/z): $[M+H]^+$ calc'd for $[C_{44}H_{52}N_2O_7]$,721.3775; found, 721.3849.

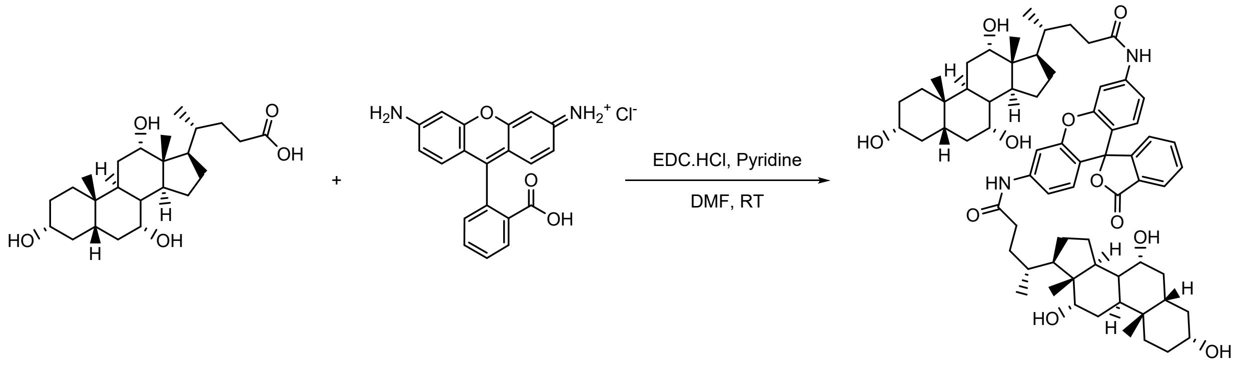


**Scheme S2. Chemical synthesis of Di-ChoRhoS.** To a dry round bottom flask, rhodamine 110 (120 mg, 0.33 mmol) and cholic acid (1022 mg, 2.5 mmol) were added. Under nitrogen atmosphere, 3 mL of dry DMF was added to the reaction mixture and left to stir for 10 min. EDC.HCl (479.3 mg, 2.5 mmol) and pyridine (3 mL) were added to the reaction mixture and left to stir at room temperature overnight. The reaction was diluted in ethyl acetate and washed with saturated sodium bicarbonate to remove extra cholic acid. The organic layer was dried using sodium sulfate and concentrated to give crude. The crude product was purified by flash chromatography (0-10%, Methanol:DCM) to obtain the final compound as an pale pink powder.

$^{1}$H NMR (400 MHz, DMSO-$d_6$) δ 10.24 (s, 1H), 8.03 (d, $J$ = 7.5 Hz, 1H), 7.87 (d, $J$ = 2.0 Hz, 1H), 7.77 (dt, $J$ = 26.7, 7.4 Hz, 1H), 7.28 (d, $J$ = 7.5 Hz, 0H), 7.18 (d, $J$ = 8.8 Hz, 1H), 6.72 (d, $J$ = 8.6 Hz, 1H), 4.33 (s, 1H), 4.22 – 3.97 (m, 2H), 3.80 (s, 1H), 3.62 (s, 1H), 3.18 (d, $J$ = 5.9 Hz, 1H), 2.36 (d, $J$ = 12.9 Hz, 1H), 2.21 (dq, $J$ = 35.0, 14.5, 11.1 Hz, 4H), 2.01 (d, $J$ = 7.9 Hz, 2H), 1.82 (dd, $J$ = 18.2, 11.2 Hz, 4H), 1.47 – 1.10 (m, 16H), 0.98 (d, $J$ = 6.0 Hz, 4H), 0.82 (s, 3H), 0.60 (s, 3H). $^{13}$C NMR (101 MHz, DMSO-$d_6$) δ 172.92, 169.18, 153.07, 152.65, 151.31, 150.55, 142.03, 136.44, 135.72, 128.78, 126.19, 125.23, 124.38, 115.67, 113.22, 106.55, 82.51, 71.46, 70.90, 66.70, 46.59, 46.22, 41.93 (d, $J$ = 14.5 Hz), 35.71 (d, $J$ = 15.6 Hz), 35.37, 34.87, 34.01, 31.81, 30.88, 29.04, 27.77, 26.69, 23.20 (d, $J$ = 18.3 Hz), 17.61, 12.84. Yield: 39 mg, 11%). ESI-LRMS (m/z): $[M+H]^+$ calc'd for $[C_{68}H_{90}N_2O_{11}]$,1111.65; found, 1111.91.

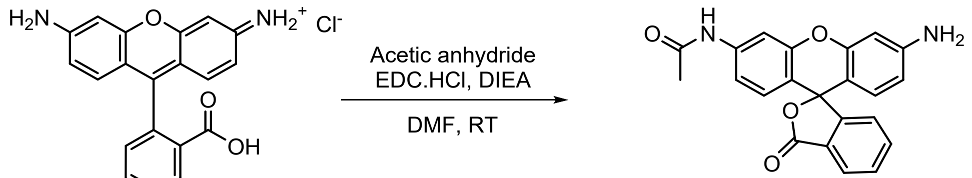


**Scheme S3. Chemical synthesis of N-acetylrhodamine.** To a solution of rhodamine (100 mg, 0.27 mmol) in anhydrous DMF (5 mL) was added DIEA (35.2 mg, 0.27 mmol) at 0 °C. Acetic anhydride (31 µL, 0.33 mmol) was added dropwise and the reaction was stirred at room temperature for 24 h, diluted with water and extracted with ethyl acetate. The organic layer was washed with water, dried with anhydrous sodium sulfate and solvent was rotary evaporated. The crude product was purified by automated flash chromatography 0-10% (methanol:ethyl acetate) to obtain the final compound as a dark orange powder (Yield, 30 mg, 30 %).

$^{1}H$ NMR (400 MHz, Methanol-$d_4$) δ 9.97 (s, 1H), 7.90 (dt, $J$ = 7.6, 1.0 Hz, 1H), 7.71 – 7.63 (m, 2H), 7.60 (td, $J$ = 7.5, 1.1 Hz, 1H), 7.14 – 7.08 (m, 1H), 6.99 (dd, $J$ = 8.6, 2.1 Hz, 1H), 6.57 (d, $J$ = 8.6 Hz, 1H), 6.47 (d, $J$ = 2.1 Hz, 1H), 6.40 – 6.29 (m, 2H), 2.04 (s, 3H). 13C NMR (101 MHz, DMSO-d6) δ 169.30 (d, J = 7.5 Hz), 152.96, 152.44, 151.84, 151.64, 141.62, 135.98, 130.45, 128.96, 128.71, 126.79, 125.01, 124.49, 115.13, 113.91, 111.72, 106.50, 105.65, 99.58, 83.95, 29.49, 24.57. ESI-LRMS (m/z): $[M+H]^+$ calc'd for $[C_{22}H_{16}N_2O_4]$,373.11; found, 373.25.

## III. **BIOLOGICAL CHARACTERIZATION OF PROBES**

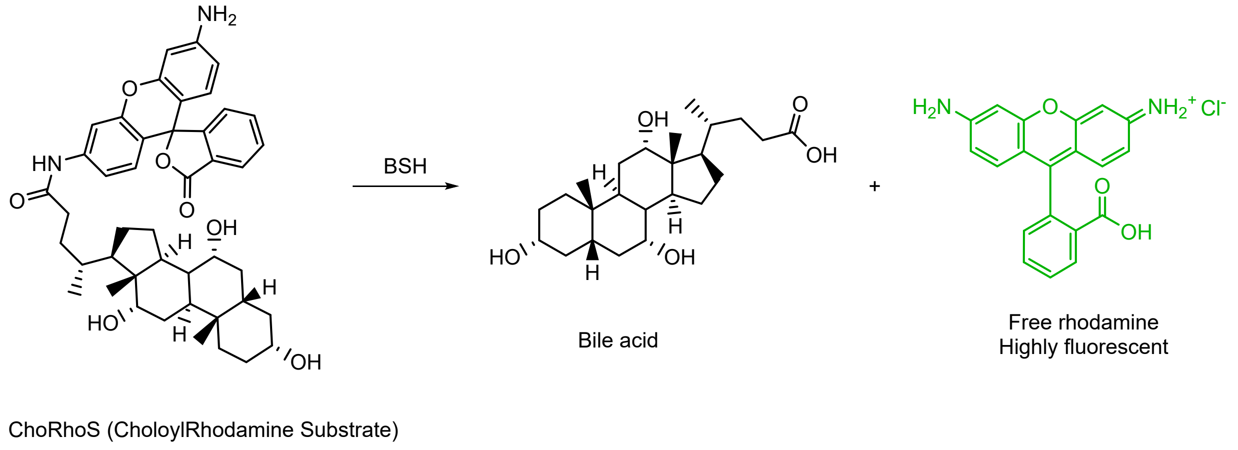


**Scheme S4. Hydrolysis of ChoRhoS.** Upon exposure of synthetic substrate to BSH, the amide bond is hydrolyzed and produces a free bile acid and free rhodamine, which is a highly fluorogenic compound compared with its conjugated version.

### Materials:

Cell lines and media: *L. plantarum* (Orla-Jensen) was purchased from ATCC (ATCC BAA-793) and cultured in MRS media (Fisher Scientific, Difco Lactobacilli MRS Broth, BD288130). *B. cereus* was purchased from ATCC (ATCC 14579). RFP *E. coli* is NEB 5-alpha F'IQ containing pGW55 (a pUC origin plasmid with KanR/Ptac:mKate2). *Both B. cereus* and RFP *E. coli* were cultured in LB media (Fisher Scientific, AC612725000) according to the vendor's recommendations.

Unless otherwise noted, all biochemical reactions were done in triplicate and the error is reported as ±SE.

**Recombinant BSH protein assay:** *L. plantarum* BSH1 was expressed and purified as previously described.[1] 50 µL solutions of probe (50 µM) in 0.1 M sodium phosphate buffer (pH 6) with 0.42% DMSO were added to 50 µL BSH (40 µg/mL) in buffer to initiate the reaction. Reaction volumes of 100 µL were used in Microfluor1 black flat bottom microtiter 96-well plates (Thermo Scientific) and the final concentration of the probe was 25 µM with 0.21% DMSO. Reactions were immediately placed in a plate reader (BioTek SynergyH1) pre-warmed to 37 °C, and reaction progress was monitored at 495 nm and 525 nm excitation and emission wavelengths, respectively, for 20 min. For Michaelis-Menten kinetics experiments, buffered solutions of 40 µg/mL purified BSH were treated with varying concentrations of substrate (0.39-25 µM). The kinetic parameters for ChoRhoS hydrolysis by BSH were determined using a Michaelis-Menten analysis.

**Whole-cell *L. plantarum* assay:** A starter culture was generated by inoculation of 5 ml MRS broth from a *L. plantarum* colony grown on MRS agar. The starter culture was incubated at 37 °C overnight. The 5 mL starter culture was then added to 45 mL fresh MRS broth and grown until the

optical density reached 0.6 at 600 nm. The bacterial culture was centrifuged at 5000 rpm for 5 min. Then the media was removed and the cell pellet was resuspended in 15 mL 0.01M PBS and centrifuged. The cells were further washed with 15 mL PBS and centrifuged once more. The resulting pellet was suspended in 1000 µL PBS. The cell suspension was then serially diluted two-fold to have different cell densities. 25 µL of each cell suspension and 25 µL 0.01M PBS buffer were added to Microfluor1 black flat bottom microtiter 96-well plates (Thermo Scientific). 50 µL solutions of probe (50 µM) in 0.01 M PBS with 0.42% DMSO were added to the cell suspensions in the plate to initiate the reaction. Reactions were immediately placed in a plate reader that was pre-warmed to 37 °C, and reaction progress was monitored at 495 nm and 525 nm excitation and emission wavelengths, respectively, for 20 min.

**Whole-cell *E. coli* plate reader assay:** A starter culture was generated by inoculation of 5 ml LB broth from an *E. coli* colony grown on LB agar. The starter culture was incubated at 37 °C overnight. The 5 mL starter culture was then added to 45 mL fresh LB broth and grown until the optical density reached 0.6 at 600 nm. The bacterial culture was centrifuged at 5000 rpm for 5 min. Then the media was removed and the cell pellet was resuspended in 15 mL 0.01M PBS and centrifuged. The cells were further washed with 15 mL PBS and centrifuged once more. The resulting pellet was suspended in 1000 µL PBS. The cell suspension was then serially diluted two-fold to have different cell densities. 25 µL of each cell suspension and 25 µL 0.01M PBS buffer were added to Microfluor1 black flat bottom microtiter 96-well plates (Thermo Scientific). 50 µL solutions of probe (50 µM) in 0.01 M PBS with 0.42% DMSO were added to the cell suspension in the plate to initiate the reaction. Reactions were immediately placed in a plate reader that was pre-warmed to 37 °C, and reaction progress was monitored at 495 nm and 525 nm excitation and emission wavelengths, respectively, for 20 min.

**Whole-cell assay with *L. plantarum* and *Escherichia coli*:** 25 µL of *L. plantarum* cell suspensions and 25 µL of *E. coli* cell suspensions at different cell densities in 0.01 M PBS buffer were added to Microfluor1 black flat bottom microtiter 96-well plates (Thermo Scientific). 50 µL solutions of probe (50 µM) in 0.01 M PBS with 0.42% DMSO were added to the cell suspensions in the plate to initiate the reaction. Reactions were immediately placed in a plate reader that was pre-warmed to 37 °C, and reaction progress was monitored at 495 nm and 525 nm excitation and emission wavelengths, respectively, for 20 min.

**Assay with mouse fecal samples:** Ice cold 0.01 M PBS with 20 mM beta-mercaptoethanol (BME) was added to fecal samples from SPF and GF mice (20 mg/mL for circadian rhythm analysis and 50 mg/mL for the rest of the experiments) and placed on ice for 30 min. The samples were extensively vortexed to obtain homogenized mixtures. After precipitation of large debris under gravity on ice, supernatant was filtered through a 35 µm cell strainer. Filtered fecal suspension was serially diluted with 0.01M PBS to test with different cell densities and 1:1 diluted fecal suspension was used for other experiments. 50 µL solutions of ChoRhoS probe (50 µM) in 0.01 M PBS with 0.42% DMSO were added to the diluted fecal suspensions (50 µL) in the plate to initiate the reaction. Reactions were immediately placed in a plate reader pre-warmed to 37°C, and reaction progress was monitored at 495 nm and 525 nm excitation and emission wavelengths respectively for 3 hours. All the reactions were done in triplicate.

For heat-shock experiments and BSH inhibitor experiments, fecal suspensions were pre-treated to lower the BSH activity with heat (95 °C) and a BSH inhibitor, caffeic acid phenethyl ester (CAPE; 200 µM), respectively, for 1 h prior to incubation with the probe.

**Human fecal sample analysis:** Human fecal samples were obtained from a healthy donor. Homogenized human fecal sample (275 mg) was suspended in ice cold 0.01 M PBS with 20 mM BME to have the initial concentration of 25 mg/mL, and serially diluted to obtain different cell densities. For sample processing and plate reader measurements, similar procedure was followed as described above for mouse fecal sample analysis.

## IV. ANIMAL EXPERIMENTS AND SAMPLE COLLECTION

**Time-restricted and *ad libitum* feeding experiments:** All the time-restricted and *ad libitum* feeding experiments were performed at Washington State University's Integrative Physiology and Neuroscience Behavioral Core. Experiments were conducted in accordance with Washington State University Institutional Animal Care and Use Committee (IACUC) protocol #6929. C57BL/6J mice were purchased from the Jackson laboratory and allowed to acclimatize to the local animal facility for 2 weeks before being used for experiments. The mice were group housed, between 2 and 3 mice per cage, and fed standard rodent chow #5001. In all experiments, 6 week-old male mice were used. Mice were 8 weeks of age at the beginning of measurements.

Mice were housed under standard 12 h/12 h light/dark conditions (light phase = 05:30 to 17:30). Time-restricted feeding groups had access to food only during the light or dark phase, respectively, for 8 consecutive days. The *ad libitum* group had access to food all day. In each group, mice (n=4) were sacrificed by $CO_2$ asphyxiation for five time points at 6 h intervals over the period of one day (starting at the beginning of the dark phase), followed by organ collection and intestine content isolation. All the samples were collected in tubes, immediately frozen in liquid nitrogen upon collection, and stored at −80 °C until analysis.

**Germ-free mouse experiments:** Mice were housed in the PNNL Animal Resource Center. All animal experiments were conducted in accordance with institutional guidelines for the care and use of laboratory animals (Battelle Richland IACUC approved protocol #2021-06). 7-8 weeks-old germ-free (GF) C57BL/6GF mice were purchased from Taconic Laboratory (German Town, NY). Male conventional C57BL/6N mice, which served as fecal microbiota transplantation (FMT) donors, were obtained from Taconic Laboratory (German Town, NY). GF mice were fed autoclaved LabDiet PMI 5010. Conventional mice were fed PMI lab Diet 5002. GF mice were group housed in a designated gnotobiotic facility room in a Tecniplast IsoP IVC caging system. Conventional mice were group housed in a separate room in Innovive IVC cages to maintain an environment similar to the GF mice. The rooms were kept at 22±2 °C and at 50±20% humidity on a 12 h/12 h light/dark cycle with *ad libitum* access to food and autoclaved water. Throughout the course of the experiment, mice stool samples were collected and tested for bacterial growth using aerobic and anaerobic culture conditions described in the next section.

GF mice were randomly divided into two groups (n=3 each), which were a control group and an FMT recipient group. The FMT group was inoculated with fecal material from conventional mice via oral gavage. After FMT, both groups were housed for 2 weeks, and mice were sacrificed by $CO_2$ asphyxiation. Intestine samples were collected in tubes, immediately frozen in liquid nitrogen upon collection, and stored at −80 °C until further analysis.

Fresh stool was collected from the male conventional C57BL/6N mice just prior to gavage. Collected fresh stool was pooled together and homogenized through vortexing in sterile 1x PBS as a 100 mg/mL slurry. The homogenized stool suspension was filtered through a 35 µm cell strainer. Mice were then colonized by oral gavage with 200 µL of filtered suspension of stool. FMT success was confirmed by culture methods identical to sterility determination for GF mice.

**Antibiotics experiments:** 5-9-weeks-old male conventional C57BL/6N mice were kept under 12 h/12 h light/dark cycle with *ad libitum* food access (PMI lab Diet 5002) at 22±2 °C temperature and at 50±20% humidity. Mice were randomly divided into two groups (n=4 each). They were given oral gavage of antibiotics (antibiotic group) or water (vehicle group) every 12 h. The

antibiotic cocktail was comprised of four antibiotics and one antifungal and was made as follows: ampicillin (100 mg/kg), metronidazole (100 mg/kg), neomycin (100 mg/ kg), vancomycin (50 mg/kg), and amphotericin B (1 mg/kg). Amphotericin B was added to prevent fungal overgrowth or opportunistic infections. This cocktail was made fresh every 36 h, aliquoted for each dose, and stored at –20° C.

## V. PROCEDURES FOR DETECTION OF BACTERIAL CONTAMINATION IN GNOTOBIOTIC MICE

Mouse fecal sample collection and handling was performed inside a clean biosafety cabinet (BSC) in a sterile manner. Feces were most commonly collected during cage changeout or other planned procedure/manipulation of germ-free mice inside the BSC. Fecal samples were collected as fresh as possible and collected directly from the anus using sterile tweezers/forceps and placed in a sterile pre-labeled screw-cap vial.

Culturing activities were performed in a clean BSC in a sterile manner. Trypticase™ soy agar with 5% sheep's blood culture plates were used for aerobic cultures, Brucella agar plates containing 5% sheep blood, hemin and vitamin K were used for anaerobic cultures, and thioglycolate growth media was used for both aerobic and anaerobic growth.

A sterile swab was premoistened with sterile 1x PBS to prevent the feces from sticking to the swab. The sample vial containing the feces was opened and the premoistened sterile swab was used to transfer a small amount of smeared sample. The thioglycolate growth media tubes were inoculated and placed in a 37 °C incubator. The same swab with feces that was used to inoculate the thioglycolate growth media was used to smear on half the surface of the blood agar plates. Using a new premoistened sterile swab, a small amount of fecal sample was transferred onto the surface of the appropriate culture plate and streaked out, spreading out over the second half of the surface of the plate. Brucella agar plates containing 5% sheep blood, hemin and vitamin K were placed in a GasPack™ EZ anaerobe gas generating pouch system, following manufacturer's instructions. The pouch containing the Brucella agar plates and the Trypticase™ soy agar with 5% sheep's blood culture plates was placed in a 37 °C incubator. The cultures were checked at 24, 48, and 72 h, and incubation was followed by 5-7 days at room temperature.

## VI. **STATISTICAL ANALYSES**

For the analysis of rhythmic oscillations, the relative fluorescence units (RFU) values for fecal samples after 84 min co-incubation with the probe were plotted against the time of collection. To analyze diurnal rhythms, the data points were subjected to cosinor analysis (i.e., fitted to a cosine function with a fixed period of 24 h) using non-linear regression.[2] Amplitude was tested against zero ($t$ test) to confirm statistical significance of rhythmicity, and peak timing was compared between experimental conditions ($F$ test) to assess phase differences.

For other analyses, pairwise comparisons were performed with Student's $t$ test using GraphPad Prism software.

VII. **SPECTRAL DATA**

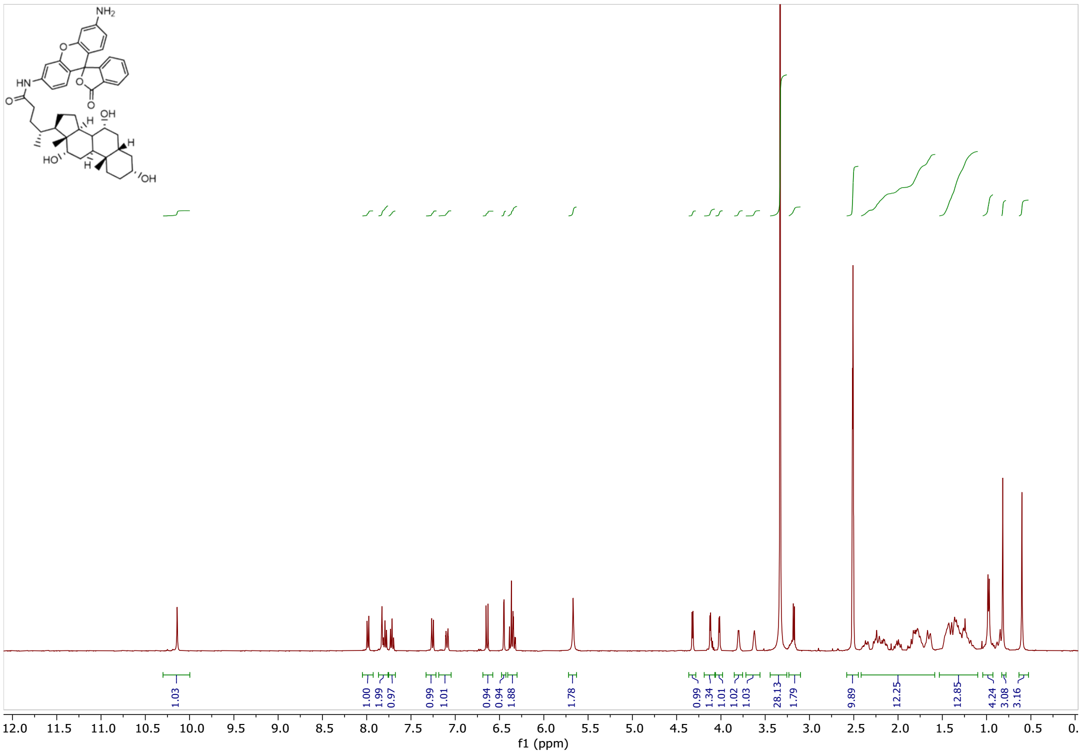


**Figure S20**. $^{1}$H-NMR spectrum for ChoRhoS.

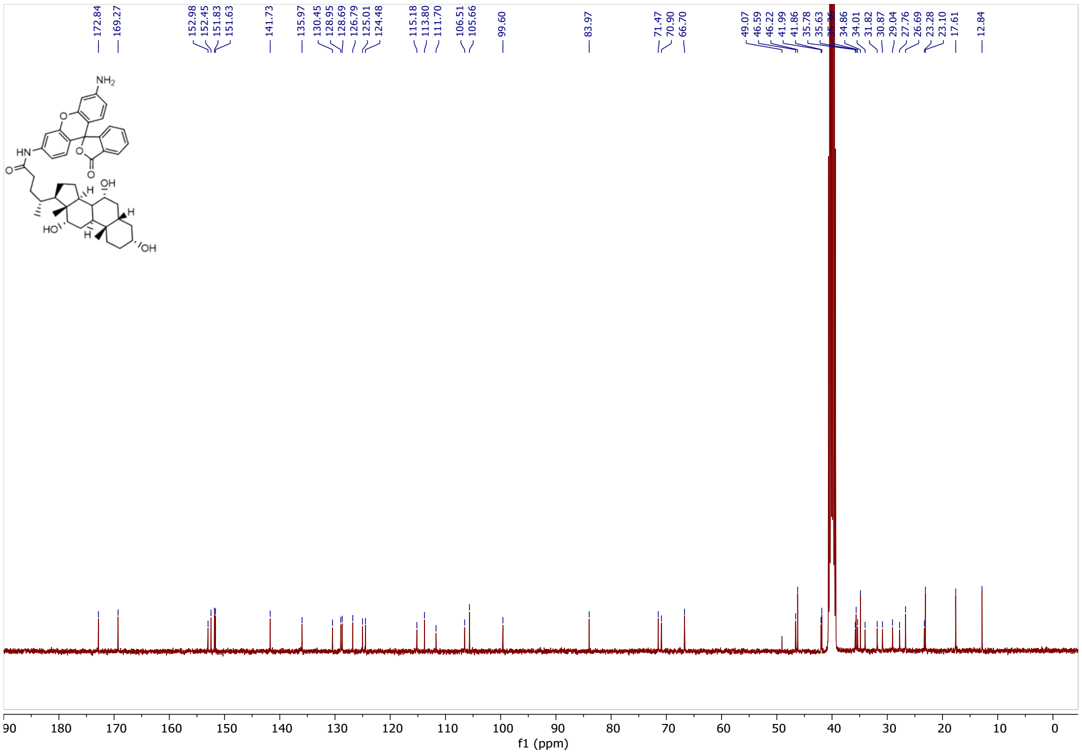


**Figure S21. $^{13}$C-NMR spectrum for ChoRhoS.**

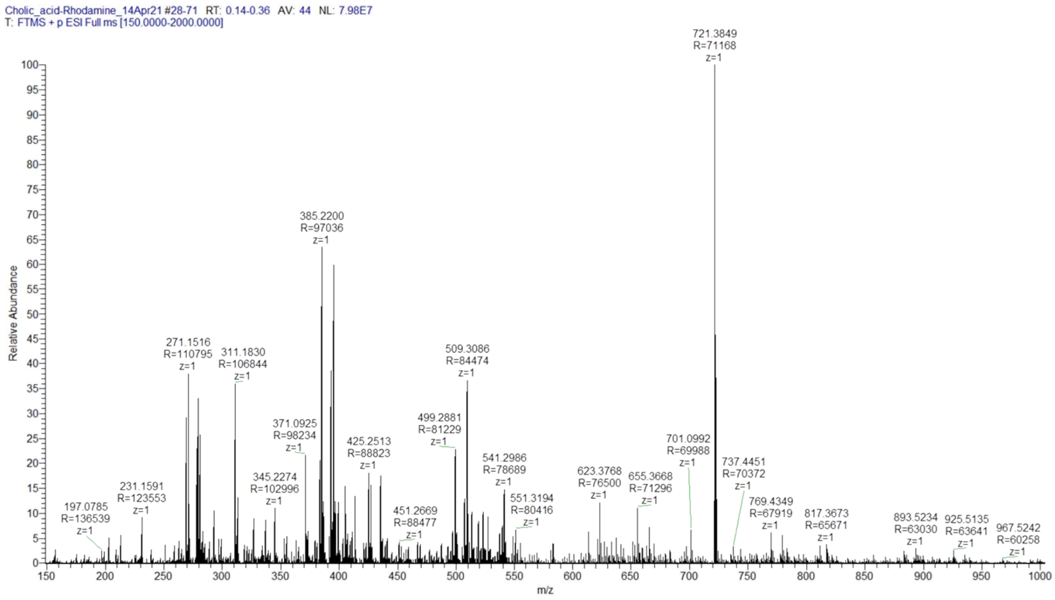


**Figure S22. HRMS spectrum for ChoRhoS.**

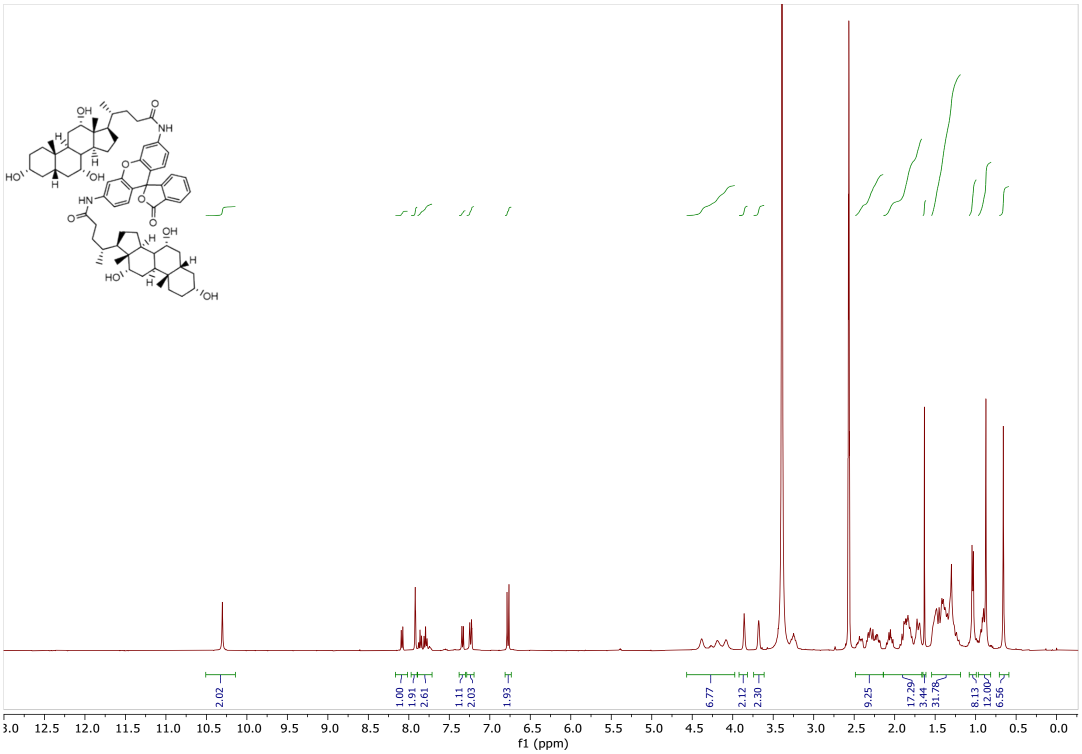

**Figure S23. $^{1}$H-NMR spectrum for Di-ChoRhoS.**

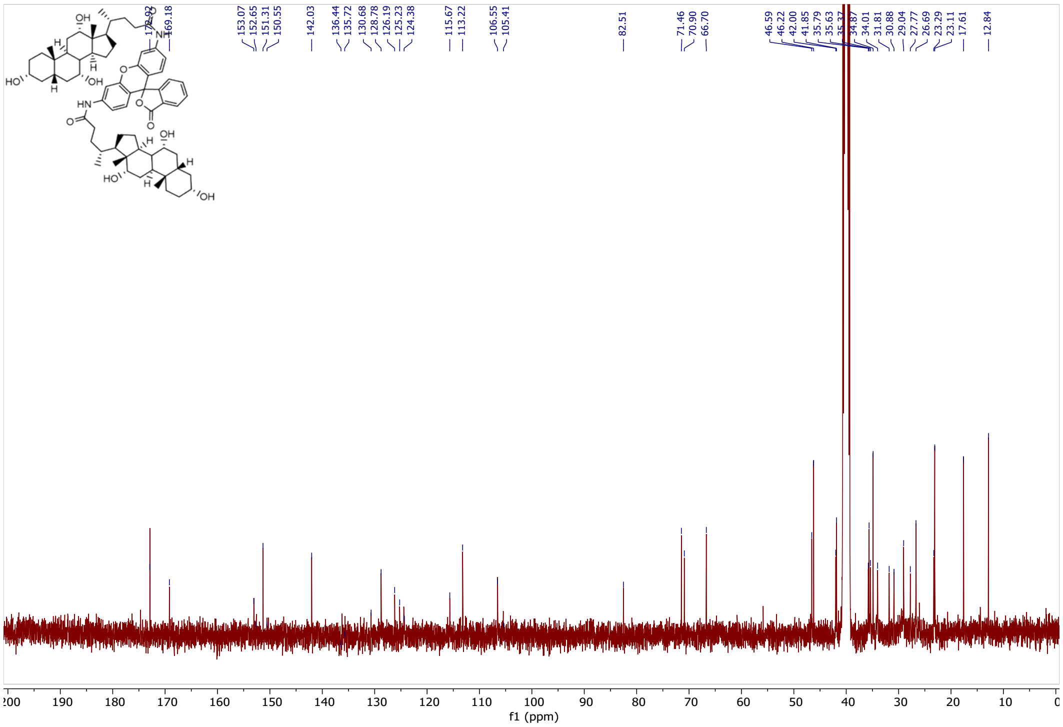

**Figure S24. $^{13}$C-NMR spectrum for Di-ChoRhoS.**

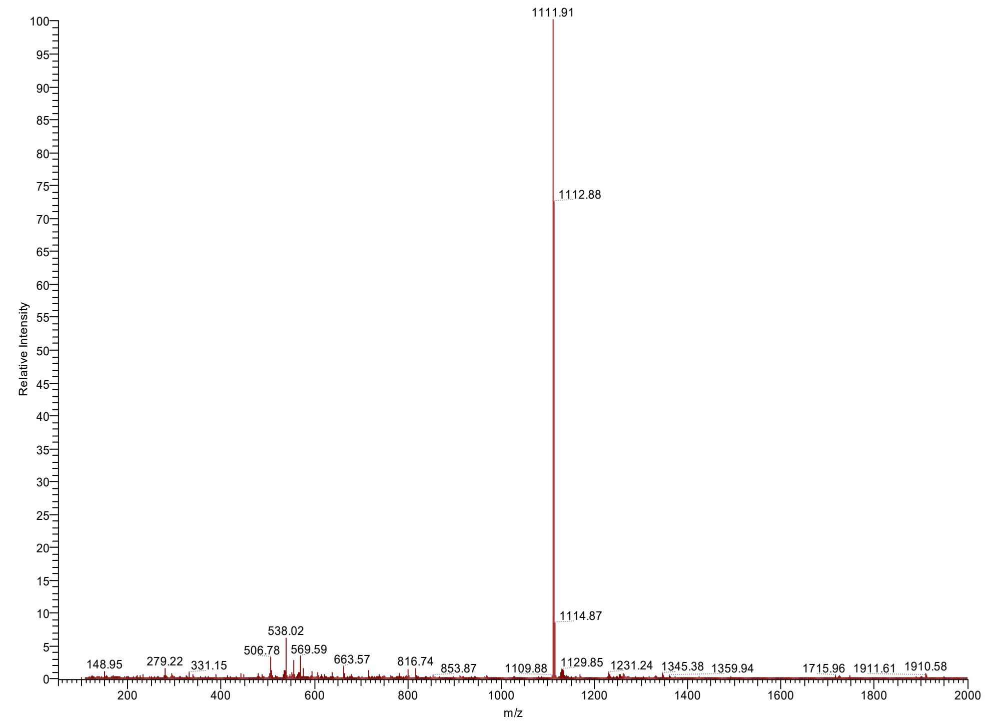


**Figure S25. LRMS spectrum for Di-ChoRhoS.**

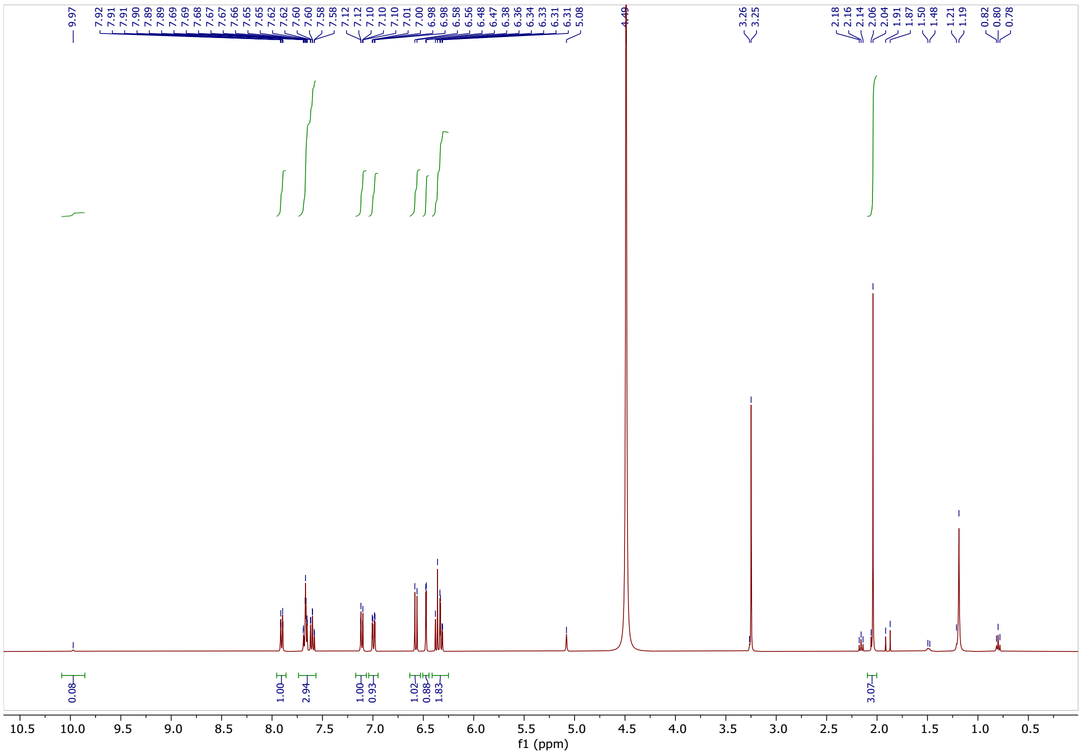


**Figure S26. $^{1}$H-NMR for N-acetylrhodamine.**

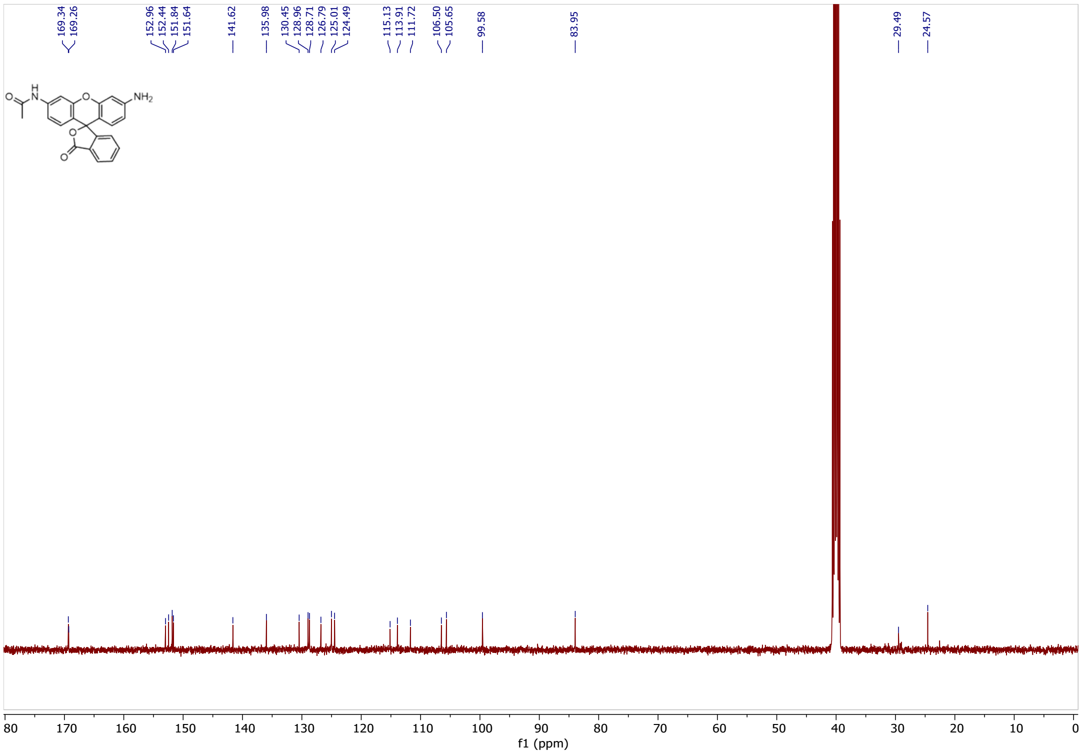


**Figure S27. $^{13}$C-NMR for N-acetylrhodamine.**

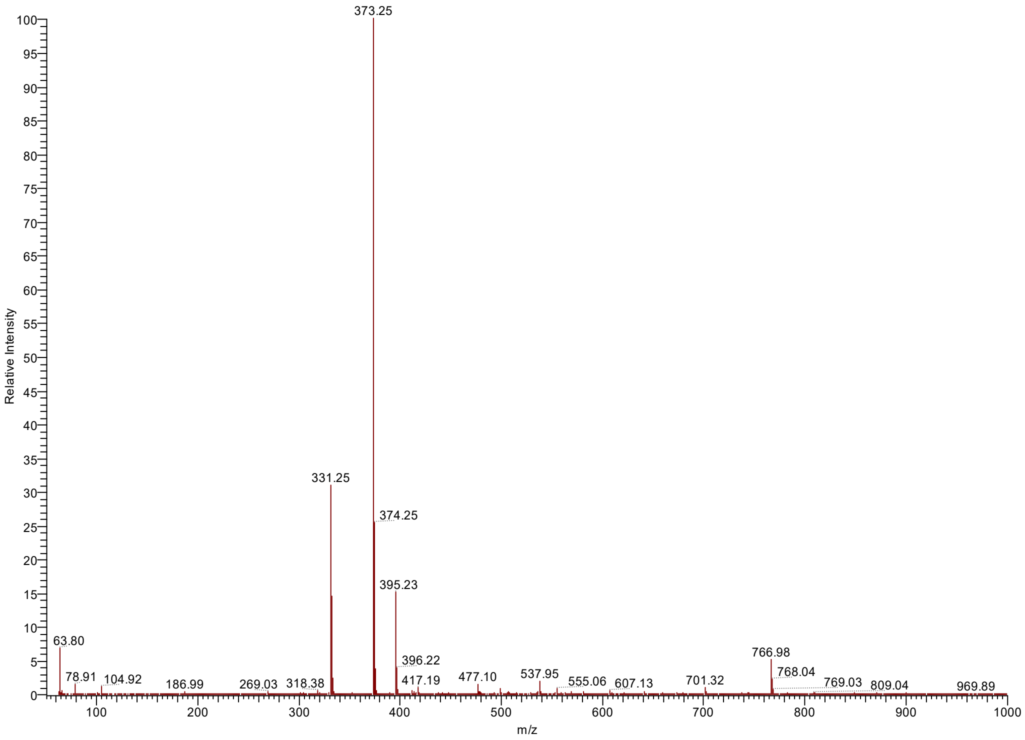

**Figure S28. LRMS spectrum for N-acetylrhodamine.**

## REFERENCES


1. Brandvold, K. R.; Weaver, J. M.; Whidbey, C.; Wright, A. T., A continuous fluorescence assay for simple quantification of bile salt hydrolase activity in the gut microbiome. *Sci Rep* **2019,** *9* (1), 1359.
2. Nelson, W.; Tong, Y. L.; Lee, J. K.; Halberg, F., Methods for cosinor-rhythmometry. *Chronobiologia* **1979,** *6* (4), 305-23.
